# Association Between Structural and Social Determinants of Health and Cognitive Functioning Among African Americans: the ARCHES Cohort

**DOI:** 10.1007/s40615-025-02730-0

**Published:** 2025-11-28

**Authors:** Jean-Francois Trani, Yiqi Zhu, Alexis I.B. Walker, Darrell L. Hudson, Ganesh M. Babulal

**Affiliations:** 1 School of Public Health, Washington University in St Louis, St Louis, MO, USA; 2 Department of Psychology, University of Johannesburg, Johannesburg, South Africa; 3 Institute of Public Health, Washington University School of Medicine, St Louis, MO, USA; 4 National Conservatory of Arts and Crafts, Paris, France; 5 Department of Health Behavior and Health Equity School, of Public Health, University of Michigan, Ann Arbor, MI, USA; 6 Department of Neurology, Washington University School of Medicine, St Louis, MO, USA

**Keywords:** Black adults, Dementia, Cognitive impairment, Multidimensional poverty, Structural and social determinants of health

## Abstract

**Background and Objectives:**

Structural and social determinants of health (S/SDOH) are linked to dementia, yet despite Black Americans having a twofold dementia risk compared to Whites, research is limited. This study examined a new S/SDOH composite index (CI) based on the multidimensional poverty-adjusted headcount ratio and explored its association with potential cognitive impairment (PCI) or poor cognitive performance (PCP) among Black Americans.

**Research Design and Methods:**

Participants were required to be age 45 or older, cognitively normal (CN), self-identified as Black or African American, and lived in the greater St. Louis, Missouri area at baseline. The S/SDOH-CI used 37 deprivation indicators from the National Institute on Aging Health Disparities Research Framework, covering environmental, sociocultural, and behavioral dimensions. Multivariate regression models were used to investigate associations between dementia and S/SDOH-CI.

**Results:**

Among 312 Black adults, 105 (33.7%) had PCI, and 47 (15.1%) had PCP. A higher proportion of those with PCI or PCP were deprived on 11 indicators (22.9% and 23.4%, respectively), compared to CN adults (13% and 15.1%). The difference in S/SDOH-CI between Black adults with PCI/PCP and CN was 62.4% and 43.9%, respectively. Key contributors of S/SDOH-CI included social factors, detrimental health behaviors, limited education, healthcare barriers, and psychological factors. The S/SDOH-CI was strongly associated with PCI (odds ratio [OR]: 11.48; 95%CI 2.08–65.00) and PCP (OR: 13.84; 95%CI 1.27–152.07) after adjusting for sex, age, and marital status. The risk was higher for Black adults over 65 (OR, 1.90; 95%CI 1.14–3.22 for PCI and OR 6.49; 95%CI 2.84–16.85 for PCP).

**Conclusion:**

These findings suggest that improving S/SDOH early in life for Black Americans through targeted public policies could reduce dementia risk later. This includes promoting equitable access to education and healthcare and implementing programs to reduce material hardship. The results also support policies that address structural racism and its impact on the health and well-being of Black Americans.

## Introduction

By 2050, the United States (US) population will increase to over 400 million citizens, and the number of older adults (age ≥ 65) will double to 88 million (20% of Americans) [1]. Over the next two decades, the population of older adults will be more racially and ethnically diverse. By 2060, increases are projected for older adults racialized as Black from 9 to 13%, Hispanic from 8 to 21%, and Asian from 4.5 to 8%, while non-Hispanic Whites (nHW) older adults will decrease from 77% to 55%. Black Americans experience poorer health outcomes as they age compared to their nHW counterparts, with a greater prevalence of chronic diseases such as hypertension, diabetes, and cardiovascular disease, as well as shorter life expectancy [2, 3]. These ethnoracial health inequities persist into older age as evidenced by an increased dementia incidence in which Black Americans have a two-fold higher risk compared to nHW [4]. Health inequities have also been substantiated by sex, particularly for Black women [5]. Furthermore, there is a higher age-adjusted risk of late onset of Alzheimer’s disease and related diseases (ADRD) for women [6]. Biomarkers alone do not explain these differences [7]. Existing studies examining racial differences in AD biomarkers via cerebrospinal fluid (CSF) have found no differences in amyloid pathology (Aβ) [8]. Additionally, older Black adults had lower rates of tau and phosphorylated tau_181_ (ptau_181_) than their nHW counterparts [9–12]. The current evidence confirms that neurogenerative diseases like AD are not spontaneous perturbations in biological processes. Rather, they are a product of the complex systems that intersect across structural and social determinants of health (S/SDOH) [13, 14]. Furthermore, these inequities stem from deeply entrenched systemic inequities, including unequal access to healthcare, socioeconomic disadvantages, and the cumulative effects of racism on physical and mental health [15].

In 2024, the updated quadrennial *Lancet* Commission on dementia prevention, intervention, and care report identified fourteen potentially modifiable risk factors for dementia, namely cholesterol, diabetes, poor education, hearing loss, hypertension, depression, smoking, excessive alcohol consumption, obesity, physical inactivity, air pollution, social isolation, traumatic brain injury, and vision loss [16, 17]. S/SDOH are the fundamental upstream factors that contribute to these conditions [18–23]. They characterize an individual’s environmental circumstances from birth until older age [24] and include micro to macro-level factors such as education, healthcare, employment, housing, food, transportation, green space, and social participation [25]. Unequal distribution of S/SDOH over the life course is influenced by socioeconomic and political power structures that have been prejudicial to minoritized groups (e.g., ethnoracial, rural, sex/gender) following a long history of racial segregation and discrimination linked to structural racism [26–28]. Structural racism and the subsequent unfair distribution of S/SDOH result in health inequities and translate into higher mortality and morbidity, shorter life expectancy, and poorer health/functional outcomes [29]. Black Americans, particularly women, have consistently experienced greater prevalence of obesity, cardiovascular and cerebrovascular diseases, psychological distress, and higher mortality [30–33], while dealing with significant barriers to healthcare access, diagnosis, treatment, and utilization [34]. The presence and severity of these barriers are associated with depression, discrimination, and stress [35], which lead to cumulative disadvantages throughout the life course and have particularly harmful outcomes in older age [36, 37]. A growing body of literature suggests that improving S/SDOH resource allocation and public health policy and education can improve outcomes [38–41]. However, historically marginalized groups continue to be differentially and negatively impacted by S/SDOH, including in the case of ADRD [42].

Few studies examine the association between S/SDOH and ADRD risk, and even fewer compare outcomes for different ethnoracial groups in the US and other high-income countries. Dimensions of deprivation reflect life course deficiencies coming from multiple domains that contribute to ethnoracial disparities in mental and physical health and increase ADRD risk at a later age. Nonetheless, the extant literature centers on single dimensions of S/SDOH and often uses race as a proxy for socioeconomic status (SES) when examining health inequities [43, 44]. For instance, results from one study indicated that older adults residing in resource-rich neighborhoods were less likely to experience cognitive decline than those in resource-poor neighborhoods [45]. Another study found that community-level S/SDOH were associated with mild cognitive impairment in an under-resourced U.S. community [46]. A third study showed an association between the Area Deprivation Index (ADI) and increased odds of ADRD neuropathology [47]. However, none of these studies investigated the intersection between race and ethnicity and multiple microsystem level S/SDOH indicators (e.g., healthcare access, education, living and working conditions, transportation) impacting the risk of dementia [25, 48]. Available indices that build on multiple indicators, such as the ADI [49, 50] and Social Deprivation Index (SVI) [51–53], conduct measurement at the census block and county-level, respectively, omitting multiple microsystem level S/SDOH, which are only measurable at the household and individual level.

The present study remediates these limitations. It establishes a new S/SDOH composite index (S/SDOH-CI) based on nine domains of the National Institute of Aging (NIA) Health Disparities Research Framework (HDRF) and a large number of indicators in order to investigate behavioral, environmental, and social risk factors of ADRD faced by marginalized populations [54]. The HDRF has been shown to be an essential resource in investigating jointly behavioral, environmental, social, and structural factors contributing to disparities in ADRD, particularly for ethnoracial minorities [55]. Previous studies have used the HDRF to investigate the complex intersection of aging and S/SDOH inequities. For instance, ambient fine particles (PM_2.5_) were associated with higher ADRD risk among Black compared to White older women [56]. Another recent study using principal component analysis found that a combination of several variables based on the HDRF domains influenced mild cognitive impairment risk, confirming that multiple and combined etiological factors explain ADRD risk [57].

We used the multidimensional poverty approach to build the S/SDOH-CI and examine how multiple S/SDOH indicators contribute together but in different proportions to ADRD risk [58]. This approach is relevant because it can show how multidimensional S/SDOH inequities may explain jointly disparities in ADRD providing unique information to guide preventive public health policies, research, and remedial programs. It has been developed to identify domains of poverty, identify disparities in poverty, highlight flaws in welfare policies that neglect certain aspects of deprivation (such as education or healthcare), and to encourage dialogue among policymakers, development actors, and researchers [59]. This “dual cut-off” approach defines poverty in two stages: first, individuals are assessed for deprivation in each dimension, with a minimum acceptable threshold determining if they are considered deprived. Then, individuals are categorized as poor or non-poor based on the number of dimensions in which they are deprived [60]. The method is widely used globally for poverty comparisons, as it incorporates a broad range of socioeconomic factors, from individual to national levels, rather than just income or utility [61, 62]. This method identifies who is deprived of S/SDOH by combining the various HDRF behavioral, environmental and social indicators and factors that impact ADRD in different socio-economic and cultural contexts [63]. It can be decomposed by subgroups around characteristics such as gender, age, ethnicity, and racial status to identify disparities in deprivation of S/SDOH. It doesn’t require a large sample size because it emphasizes identification and intensity of deprivations, is based on categorical data, allows disaggregation, and avoids statistical assumptions about the distribution of variables that require large sample. Recent studies have used multidimensional poverty measures to link various S/SDOH with health status and behavior [20], chronic health conditions [64], disability [65], and ADRD [66–68].

Our study investigated the relationship between the S/SDOH-CI and cognitive impairment in a sample of middle-to-older age Black adults living in the greater metropolitan St. Louis area. Our research questions are as follows: (1) Whether higher multidimensional S/SDOH burden is associated with prevalent cognitive impairment at enrollment; (2) whether this association persists after covariate adjustment; (3) the robustness of findings across alternative index specifications; and the contribution of each indicator and factor to the overall deprivation of S/SDOH. The study question is: what is the association between S/SDOH and cognitive impairment? We hypothesized that (1) the S/SDOH-CI encompassing deprivation in multiple HDRF domains, factors, and indicators would be positively associated with poorer cognitive functioning among adults racialized as Black, (2) the association would be higher for older women confirming existing evidence [69, 70], (3) and highest contributing factors would be limited education [71], neighborhood disorders [72, 73], poverty [74, 75], discrimination [25], stress [76–78], and substance use [79].

## Design and Methods

### Study Design and Sample

Aging Research Characterizing Health Equity via Social Determinants (ARCHES) cohort study examining biological and S/SDOH among Black Americans initiated recruitment March 31, 2022 with a planned sample size of 300 participants age 45 years or older, self-identified as Black or African American, residing in the greater St. Louis region and cognitively normal (CN) based on the blind Montreal Cognitive Assessment (MOCA) screening completed over the phone [80, 81]. As of November 2024, 312 participants were enrolled. All participants completed a cognitive assessment, an S/SDOH battery using scales reflecting the various dimensions of the HDRF, a blood draw, and an MRI at their enrollment visit.

### Cognitive Functioning

Participants completed a combination of neuropsychological tests and clinical assessment. Potential cognitive impairment (PCI) was examined using the MOCA, and a cut-off was set at a score of ≤ 23 [82]. A second, more robust assessment, the preclinical Alzheimer’s cognitive composite (PACC), examined several cognitive domains, including episodic memory (Selective Reminding test Free and Cued Recall) [83], attention control (Trail Making Test A), executive control (Trail Making Test B) [84], and verbal fluency (Animals Naming) [85]. Trail-Making Test A and B scores were reverse-coded to ensure that a higher score indicated better cognitive performance. The PACC score was computed by averaging the z-score across the measures [86]. A score of 1 SD below the mean was considered poor cognitive performance (PCP).

### Dimensions, Factors, and Indicators of S/SDOH

We identified ten factors across three dimensions of the HDRF—environmental, sociocultural, and behavioral. We operationalized these dimensions via validated instruments with standardized deprivation thresholds (typically ≤ − 1 SD for deficits and ≥ + 1 SD for adverse exposures). The environmental dimension captured neighborhood social cohesion/exchange (deprivation ≤ − 1 SD) and physical disorder (≥ + 1 SD); education quality, defined as no college attendance and the Wide Range Achievement Test reading ≤ − 1 SD [87]; household living standards (perceived conditions, transportation, communication, and an asset index). For living standards, deprivation was defined as non-homeownership with overcrowding (> 2 persons/room), lack of car access, or no internet; the asset index was constructed using polychoric principal component analysis, with deprivation defined as ≤ − 1 SD on the first component [88]. Barriers to care were assessed with a 17-item Barriers to Care Scale spanning nine components [89]; participants were classified as deprived within a component when endorsing predefined difficulty thresholds.

The sociocultural dimension included religiosity (Duke Religion Index subscales ≤ − 1 SD) [90], social hardship (any reported difficulty meeting basic needs), and life stressors (≥ + 1 SD on the 8-item Ongoing Chronic Stressors scale [91]. Psychological characteristics were measured using the 50-item International Personality Item Pool, with deprivation defined as ≤ − 1 SD in extraversion, agreeableness, openness, or conscientiousness, or ≥ + 1 SD in neuroticism [92]. Life satisfaction was assessed with the SWLS, with deprivation defined as ≤ − 1 SD [93].

The behavioral domain comprised high-effort coping (John Henryism ≥ + 1 SD) [94], vigilance to discrimination (Heightened Vigilance ≥ + 1 SD) [95, 96], perceived everyday discrimination (≥ + 1 SD) [97], and seven health behavior risks (e.g., current smoking, alcohol/substance use, physical inactivity, food insecurity) with indicator-specific cutoffs [98, 99].

Collectively, the SDOH-CI integrates neighborhood and household conditions, education and healthcare access barriers, sociocultural milieu, psychological traits, and health behaviors to capture multilevel risks relevant to cognitive aging and ADRD (See details in the Supplementary Tables 1 and the online resource). It comprises 37 indicators, proxies of S/SDOH that influence critical outcomes, including health, disability, functioning, and quality of life.

### Existing Indices of Vulnerability and Deprivation

SES and neighborhood quality are important factors influencing health and can be measured to evaluate their impact [100]. Poor neighborhoods are sources of low employment opportunities, remote green space, poor education quality, lack of healthy food options, discrimination, structural racism, and limited healthcare access [101, 102]. The ADI ranks neighborhood disadvantage based on 17 factors, including education, employment, and housing, indicators from the US Census Data [103]. Similarly, The Centers for Disease Control and Prevention and Agency for Toxic Substances and Disease Registry Social Vulnerability Index (hereafter, CDC/ATSDR SVI or SVI) uses 16 US census variables to construct a vulnerability index based on social, economic status, household character, ethnoracial compositions of the communities, and household styles and transportation [104]. We used study participants’ Federal Information Processing Standard (FIPS) codes to derive their corresponding ADI and SVI.

### Statistical Analysis

We assessed deprivation of S/SDOH across different dimensions, factors, and indicators of the HDRF, comparing study participants’ cognitive status using the Alkire and Foster (2011) multidimensional poverty approach, which identifies dimensional shortfalls. Dimensions of deprivation were independently assessed through one or multiple indicators. The method considers two cut-offs. A participant was identified as deprived on one indicator if they fell below the cut-off score determined for that specific indicator. The second cut-off *d* was determined by the number of weighted indicators in which a participant must be deprived to be deemed overall poor regarding S/SDOH. We set *d* = 11 indicators corresponding to a prevalence of deprivation of S/SDOH in our sample close to 19.2%, corresponding to the proportion of older Black adults over 65 deemed poor in the U.S [105].

One-way ANOVA analyses examined disparities in level S/SDOH indicators between cognitively normal participants and those who were PCI and PCP and compared sex and age groups. We adjusted for post-hoc pairwise comparisons [106]. We also carried out Spearman rank correlation analysis to assess the overlap of S/SDOH indicators.

We calculated three S/SDOH poverty measures: (i) the headcount ratio (H) which indicates the number of adults who were disadvantaged on d = 11 S/SDOH indicators; (ii) the average S/SDOH deprivation share (A), which is the average number of poor S/SDOH indicators endured by each adult deemed disadvantaged of S/SDOH; and (iii) the adjusted headcount ratio (M0), which is the product of the headcount ratio (H) and the average S/SDOH deprivation share (A); (M0) denotes the incidence of multidimensional S/SDOH deprivation and corresponds to the unidimensional S/SDOH-CI for d = 11. We used the mpitb R-package for multidimensional poverty measures.

Robustness was examined via two different weighting structures: (i) Equal weight of one for every indicator (equal indicators weight [EIW]) in each HDRF factor; (ii) Equal weight associated with the ten HDRF factors and equal weight for each indicator within each factor (Equal nested weight [ENW]). In a factor with one indicator (e.g., coping), the indicator’s weight was one. But the weight of each indicator was 1/3 in a factor with three indicators (e.g., religiosity). Both methods provided consistent results.

The contribution of each indicator was determined by the product of the number of study participants deprived on that indicator by its weight, divided by the product of the number of study participants deprived in 11 indicators or more and their respective weights [60]. The contribution of each indicator was combined into factors. Factor contributions were calculated for all study participants and compared according to cognitive status, sex, and age. R-package MPI was used to calculate the level of deprivation and dimension contribution of factors [107].

We calculated unadjusted and adjusted logistic regression models to identify the association between PCI and PCP and multidimensional S/SDOH deprivation. The binary outcome compared study participants with and without cognitive impairment, whichever scale was considered. The individual burden of poor S/SDOH was calculated by dividing the weighted indicators on which this individual was deprived by the total weighted indicators among individuals deprived of 11 indicators or more (i.e., the cut-off for multidimensional S/SDOH deprivation). We conducted a secondary set of analyses that supplanted the S/SDOH-CI with the ADI and SVI separately. We adjusted for sex (male/female) and age (below 65/65 and above). A p-value of < 0.05 was considered significant. Generalized linear regression package in R was used to conduct regression analysis.

## Results

### Participants Characteristics

Overall, 312 individuals with complete data were included. Our enrolled sample is older (65 + years: 51.7% vs. 17.3% nationally), includes fewer men (22.4% vs. 49.6%), and has a higher proportion with a bachelor’s degree or higher (52.9% vs. 34.3%). Participants, on average, reside in similar level of disadvantaged neighborhoods (mean sample ADI: 75.0/100 vs. national ADI 75.6/100).

Based on the MOCA, the distribution was 105 participants with potential cognitive impairment (PCI) and 208 cognitively normal (CN). Based on PACC, there were 47 participants with poor cognitive performance (PCP) and 266 CN. There was a lower proportion of women with PCI and PCP (32.6% and 11.6%) than men (37.1% and 27.1%) in our sample. The mean age of adults with PCI and PCP was significantly higher than those who were CN (Table 1).

**Fig. 1:**
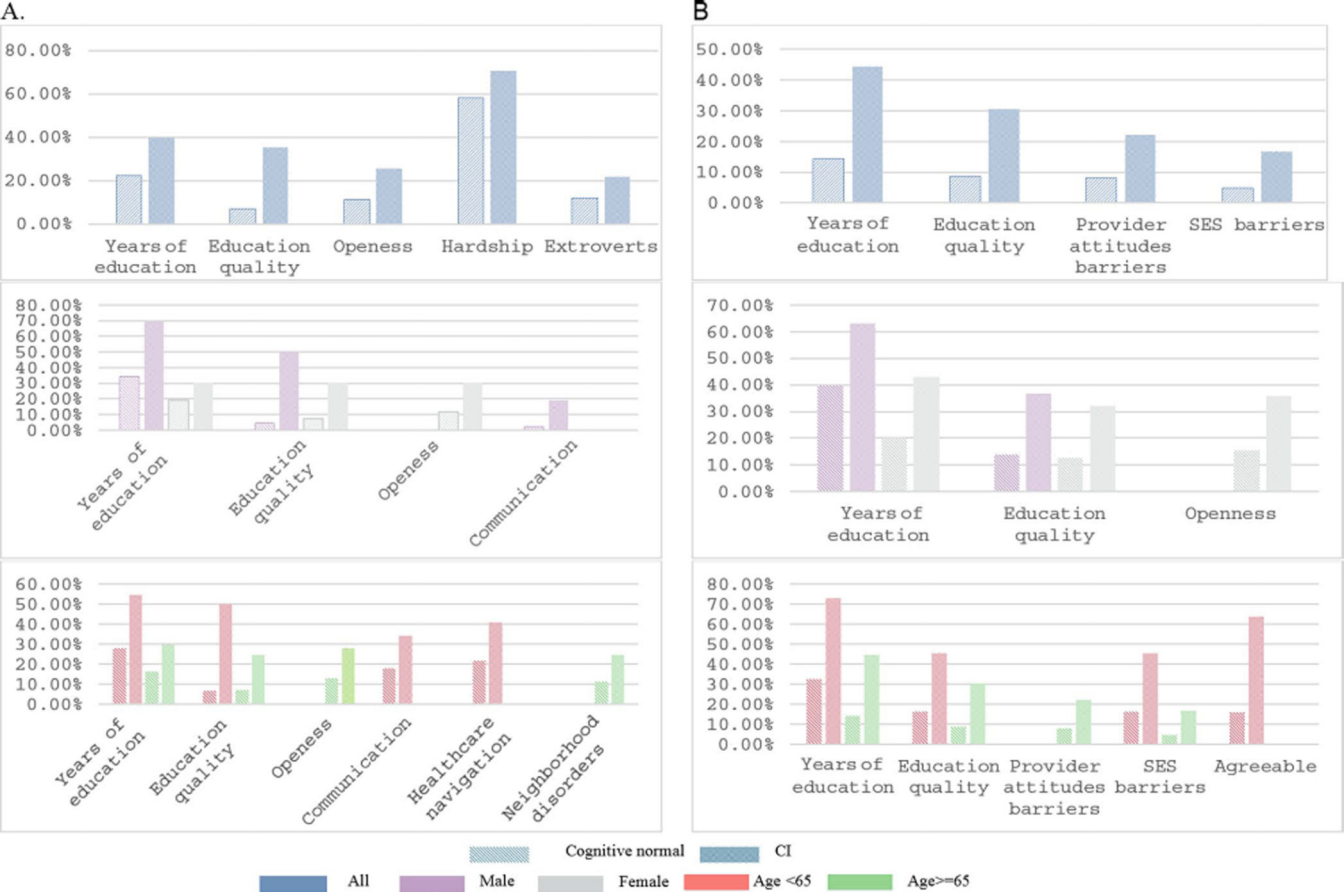
The significant difference in the percentage of deprivation between CN and CI

A higher number of Black adults with PCI were deprived on seven indicators compared to CN adults, namely: Neighborhood disorder (10.8% gap, *p* = 0.028), years of education (17.8% gap, *p* = 0.002), reading skills (28.4% gap, *p* < 0.001), hardship (12.5% gap, *p* = 0.042), extraversion (9.8% gap, *p* = 0.035), openness (14.6% gap, *p* = 0.002), and neuroticism (10.3%, *p* = 0.034). Similarly, Black adults with PCP experienced significantly more deprivation on four indicators, namely: years of education (30%, *p* < 0.001), reading skills (21.8%, *p* = 0.002), healthcare barriers due to provider attitudes (14.2%, *p* = 0.037), and socioeconomic status (11.9%, *p* = 0.045). The gap between PCP and CN was not significant for hardship, extraversion, and neuroticism (Fig. 1 and Supplementary Table 2).

Black men with PCI and PCP were less likely to attend private religious activities compared to their CN counterparts. Similarly, Black adults with PCI and PCP, across all age groups, were more deprived in education, both in terms of years completed and reading capacity, compared to CN. Among Black women, those with PCI and PCP experienced more deprivation in personality traits, particularly openness, than those in the CN group. In contrast, Black men with only PCP were more deprived in neuroticism than CN men.

Black adults under 65 with PCI and PCP were more likely to face deprivation in transportation access and healthcare navigation compared to CN. Additionally, barriers to healthcare access linked to SES, food insecurity, and lower levels of agreeableness were significantly more prevalent among Black adults with PCP under 65 compared to CN. For adults aged 65 and above, those with PCI showed significantly more deprivation in personality traits, specifically neuroticism, conscientiousness, and openness, compared to their CN peers in the same age group.

Spearman-rank correlation coefficients between each pair of deprivation indicators found no evidence of a strong correlation between indicators (Supplementary Table 3). This absence of overlap between indicators validates a multidimensional approach that does not rely on one or few indicators of S/SDOH deprivation but represent all aspects of S/SDOH deprivation.

### Incidence of Multidimensional S/SDOH Deprivation

Deprivation of S/SDOH varied among study participants based on sex, age, and cognitive status, depending on the number of indicators considered. We found that 99.1% of Black adults with PCI and 99.7% with PCP were deprived in at least one indicator. In contrast, no participant was deprived across all 10 factors and 37 indicators. Only 1% of Black adults with PCI and 2% with PCP in our sample were deprived on 23 indicators, with none exceeding that number. For d = 11 indicators (i.e., the cut-off for multidimensional S/SDOH deprivation), the proportion of participants considered deprived of S/SDOH was 22.9% for Black adults with PCI and 23.4% for those with PCP, compared to 13.0% and 15.1% for CN Black adults (Supplementary Table 4).

**Fig. 2:**
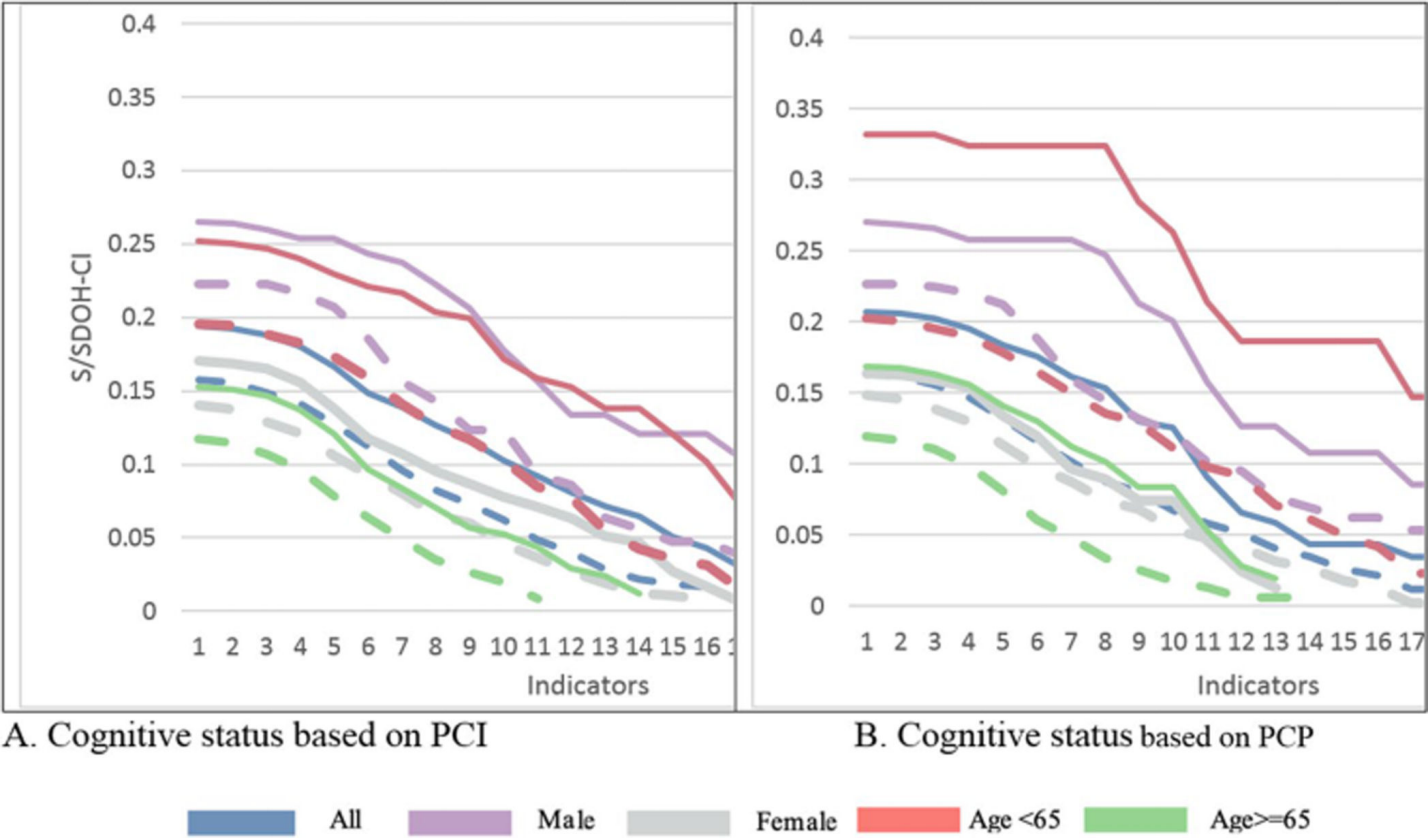
S/SDOH-CI by cognitive status according to MOCA, PACC, age group, and gender. Notes: PCI: Continuous line is used for people cognitively impaired and dash lines for people cognitively normal; possible cognitive impairment as measured by the Montreal Cognitive Assessment (MoCA); PCP: poor cognitive performance as measured by the preclinical Alzheimer’s cognitive composite (PACC).

Black adults with PCI and PCP experienced a significantly higher incidence of S/SDOH deprivation (as measured by M0) compared to CN adults, regardless of the cut-off value (d), ranging from 1 to 19 (Fig. 2 and Supplementary Table 4). The difference in S/SDOH deprivation ranged respectively from a minimum of 20.7% and 23.6% for d = 1 to a maximum of 116.9% and 95.9% for d = 17, for Black adults with PCI and PCP compared to CN Black adults. S/SDOH deprivation was significantly higher at most cut-offs for both Black women and men with PCI and PCP, when compared to their CN counterparts (Fig. 2 and Supplementary Table 4).

Differences in multidimensional S/SDOH deprivation, increases with the cut-off (d). For *d* = 11, the S/SDOH-CI was 62.42% higher among Black adults with PCI compared to CN (*p* = 0.009). Significant differences were also observed in the S/SDOH-CI between Black women with PCI (64.4%, *p* = 0.026), Black adults under 65 with PCI (60.6%, *p* = 0.017), and Black adults over 65 with PCI (132.4%, *p* = 0.006) compared to CN. However, no significant difference was found among Black men.

For Black adults with PCP, the S/SDOH-CI was 43.9% higher than CN Black adults, but this result was only marginally significant (*p* = 0.088). Significant differences were noted within age groups: a 120.7% increase for Black adults over 65 and a 74.03% increase for those under 65, compared to CN. No significant differences were found between Black women and men with PCP relative to CN (Supplementary Table 4).

### Indicators Contributing Most To Multidimensional S/SDOH Deprivation

**Fig. 3:**
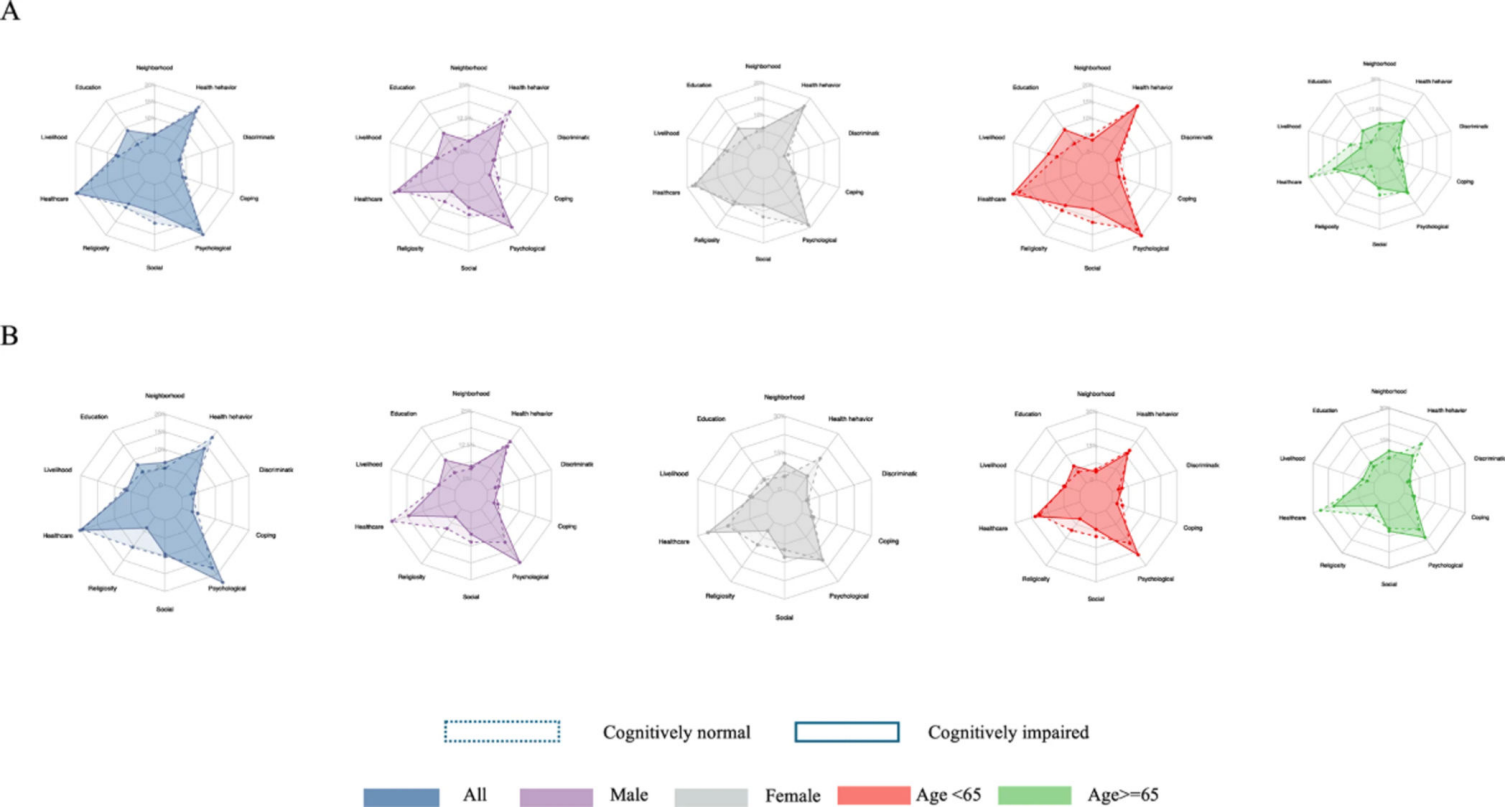
The radar webs of factors contributions to the S/SDOH-CI. Notes: A: The difference in dimensional contributions to the S/SDOH-CI between cognitive normal Black adults and those with possible cognitive impairment (PCI); B: The difference in dimensional contributions to the S/SDOH-CI between cognitive normal Black adults and those with poor cognitive performance (PCP)

Each indicator contributed to multidimensional S/SDOH deprivation across different cutoff levels (*d*). For *d* = 1 to *d* = 13, the most significant contributors to multidimensional S/SDOH deprivation, irrespective of cognitive status, sex, or age group, included hardships related to food, housing, rent payments, and illicit drug use (data not shown). Radar graphs in Fig. 3 illustrate the contributions of 37 indicators grouped into the 10 HDRF factors to the S/SDOH-CI for *d* = 11. The shapes of the colored areas in the radar graphs illustrate the percentage contribution of each factor, emphasizing those most contributing to multidimensional S/SDOH deprivation. Overlapping areas highlight similarities in contributions between CN and PCI/PCP subgroups. Contributions to the S/SDOH-CI vary based on cognitive status and demographic characteristics such as sex and age. Cognitive status differences are represented by lighter, dashed-colored areas (cognitively normal) and darker, solid-colored areas (cognitively impaired), while distinct colors denote variations by sex and age. The top four contributing factors were detrimental health behaviors, personality traits (the psychological factor in the HDRF), education, and healthcare access.

Six indicators of detrimental health behaviors collectively accounted for 16.2% of the S/SDOH-CI for PCI, 13.9% for PCP, and 17.6% for CN. These contributions were higher for women and adults over 65 in the CN group compared to PCP and higher for men in the CN group compared to PCI. Deprivation in the five personality traits—extraversion, agreeableness, openness, conscientiousness, and neuroticism accounted for 17% of the S/SDOH-CI for PCI, 20.3% for PCP, and 13% for CN. Including life satisfaction increased contributions to over 20% for PCI and PCP, compared to 17% for CN, with no significant differences by sex or age for PCI. Barriers to healthcare contributed 19.8% to 20.3% of the S/SDOH-CI for PCI and PCP, comparable to 19.5% for CN across all demographics. Education and reading capacity levels contributed 8.9% and 8.2% to the S/SDOH-CI for Black adults with PCI and PCP, nearly double the contribution seen in CN for both sexes and age groups.

Other factors contributed less. Three indicators of religiosity contributed around 10% to CN, 7.8% to PCI, and 3.8% to PCP, with consistent differences across demographics except for PCI women, where religiosity contributed slightly more than for CN women. Daily stressors and hardships (the social factor in the HDRF) contributed jointly about 10%, slightly more for CN than PCI or PCP, except for women and older adults (over 65) with PCP, where contributions exceeded those for CN. The coping factor combining John Henryism and vigilance contributed less than 5% to the S/SDOH-CI, regardless of cognitive status, sex, or age group (Supplementary Table 5).

### Multivariate Analysis

The relationship between the individual burden of poor S/SDOH and the risk of PCI and PCP was examined. As shown in Table 2, the risk of PCI was significantly higher for adults with a high burden of poor S/SDOH, being 6.69 times greater (95% Confidence Interval [CI], 1.43–31.70, *p* = 0.016) in the unadjusted model, and 11.48 times greater (95%CI, 2.08–65.00.08.00, *p* = 0.005) after controlling for sex, age, and marital status. Female sex did not contribute to an increased relative risk of PCI, but age was a significant factor, with the risk being 1.91 times higher (95%CI, 1.14–3.23, *p* = 0.014) for adults aged 65 and older compared to those under 65.

For PCP, the risk was not significantly higher in the unadjusted model, but it was 13.84 times higher (95%CI, 1.27–152.07.27.07, *p* = 0.030) in the adjusted model. Both age and male sex were associated with increased odds of PCP. Being married or living with a partner did not significantly protect against either PCI or PCP. The associations between the ADI and the SVI with the risk of PCI and PCP were also explored (Supplementary Table 6a and 6b). Neither PCI nor PCP was significantly associated with the ADI. However, a slight increase in risk was found with the SVI: the risk for PCI was 1.14 times higher (95%CI: 1.01–1.25, *p* = 0.027), and for PCP, it was 1.15 times higher (95%CI: 1.00–1.31.00.31, *p* = 0.041). In the adjusted model, the association for PCI remained significant (OR: 1.13, 95%CI: 1.01–1.25, *p* = 0.027), but for PCP, it was not (OR: 1.12, 95%CI: 0.97–1.29, *p* = 0.109).

### Sensitivity Analysis

Measures of S/SDOH deprivation were recalculated using the equal-nested weight (ENW) structure based on the 10 HDRF factors. The results consistently showed similar patterns when broken down by sex and age groups. The S/SDOH-CI was significantly higher for older Black adults with PCI and PCP than CN, at a 30% cut-off across all indicators (*p* < 0.05). Black adults with PCI and PCP exhibited a notably higher S/SDOH-CI than CN across all sex and age groups for this same cut-off.

Contributions of various dimensions to the S/SDOH-CI consistently highlighted the dominance of social factors (including hardship), education, detrimental health behaviors (although drug use was less significant than food insecurity), and psychological factors in driving poverty for both older adults with PCI and PCP, as well as for CN, across both sexes and age groups. The coping factor, particularly vigilance, was found to contribute more than in the EIW structure.

## Discussion and Implications

This study highlights significant inequities in S/SDOH among older Black adults with possible cognitive impairment (PCI) and poor cognitive performance (PCP) compared to cognitively normal (CN) adults. We found that a higher S/SDOH-CI score, built on 37 indicators, within ten factors across three domains—environmental, sociocultural, and behavioral determinants of health— defining the National Institute on Aging Health Disparities Research Framework (NIA/HDRF) was associated with PCI and PCP. These factors, which include neighborhood, education, living standards, healthcare, religiosity, social and life stressors, psychological well-being, coping, discrimination, and health behaviors, provide a broader view of S/SDOH compared to commonly used indices in dementia research, such as the ADI and SVI.

Regardless of sex or age group, older Black adults with PCI and PCP were found to experience more S/SDOH deprivations than CN adults. The difference in S/SDOH-CI was significant for older Black adults with PCI and marginally below the *p* < 0.05 threshold for those with PCP, considering a cut-off of 11 indicators. If the cut-off was adjusted to 10 indicators reflecting a poverty rate just above the US poverty line for older Black adults, the differences were significant for both groups. Interestingly, the risk of PCI and PCP associated with higher S/SDOH-CI scores was not higher for women, which contrasts with previous research suggesting male sex may be a protective factor against dementia [108]. Additionally, living with a partner was not significantly associated with a reduced risk of PCI or PCP. On the other hand, age was found to be a significant risk factor for both PCI and PCP.

Furthermore, these findings suggest that factors of deprivation in S/SDOH were substantially higher for older Black adults with PCI and PCP compared to CN adults. First, deprivation of education was higher for both older Black women and men with PCI and PCP of all age groups, confirming that education is an important protective factor for dementia [16, 109]. Second, deprivation in quality and affordable healthcare was also more pronounced among the former, reaffirming the call for better access and participation in ADRD research for racial and ethnic minorities. Social stressors–daily stressors and hardship–Black adults undergo during their life course were also more prevalent for PCI and PCP, confirming that stress is a significant mechanism that fuels Black-White health inequities in the US, thus increasing the allostatic load of the body and weathering [110]. Finally, extraversion and openness were also higher among personality factor indicators for older Black adults with PCI and PCP than CN. To date, extraversion appears unassociated with dementia risk, while openness has been considered a possible protective factor against dementia, primarily among nHW samples [111, 112]. However, this pattern may differ for Black Americans, confirming an interaction between structural racism, individual and community circumstances, and health outcomes [7]. Present and historical structural racism nurtures racial discrimination in all aspects of S/SDOH during the life course, including housing, education, healthcare, socioeconomic circumstances, and socialization, explaining health inequities, poor health outcomes, including ADRD among ethnoracial minorities [27, 113].

These findings confirm the central role of inequities in resource allocation in S/SDOH on ADRD risk. Alleviating these inequities could prevent ADRD or slow down cognitive decline in minoritized communities. Individual-level interventions alone have a limited impact on preventing ADRD risk. Public policies addressing upstream and distal factors, providing access to quality education and healthcare, fighting stigma and discrimination to reduce stress, and funding welfare programs to reduce material hardship for the poorest households may reduce and even suppress risk factors for ADRD later in life. Additionally, we made the case for a new intersectional measure of S/SDOH to unpack the subtlety and complexity of the relationship between various environmental factors and dementia risk during the life course. We introduced a distinctive composite index that operationalizes the multidimensional S/SDOH deprivation by combining multiple environmental factors influencing people’s life course into a unidimensional measure and examining its link with ADRD risk. Alternative measures of SES used in the literature, such as the ADI and the SVI, are more limited in their intersectionality of factors, offering a less accurate appraisal of this relationship [114]. Hence, the association with dementia risk was not significant.

To account for cultural values, lived experiences, and community contexts, we argue here that interventions and programs should be co-designed with community partners and delivered through community-anchored venues—for example, local anchor institutions and other high-trust community environments. They should use culturally concordant navigators, tailor communication to language and literacy, incorporate trauma-informed, stigma-aware practices, and monitor acceptability with culturally validated measures. Culturally concordant navigators—including community health workers and peer/patient navigators— may improve screening, treatment timeliness, chronic disease control, and retention in care among underserved populations, as shown in recent systematic reviews across cancer care and multiple conditions [115]. Peer navigation specifically improves initiation and retention in HIV care, underscoring the generalizability of navigator models to equity-focused programs [116]. Language concordance between staff and health program participants has been associated with better communication and outcomes [117]. Incorporating trauma-informed, stigma-aware practices enhances engagement and psychosocial outcomes in health settings, supporting their inclusion in navigator training and protocols [118]. Finally, routine monitoring of acceptability with validated instruments aligns with implementation science principles of acceptability, appropriateness, feasibility, providing a clear framework for measuring uptake and fit in culturally responsive interventions [119].

This study has some limitations. First, the cross-sectional nature of the data makes it challenging to establish the direction of causality between multidimensional S/SDOH deprivation and PCI and PCP. Multidimensional S/SDOH deprivation could be a cause or an effect of dementia. Future research should follow this cohort to establish time-dependent relationships and unpack causal mechanisms between S/SDOH and ADRD risk. Second, the limited sample size makes it difficult to investigate further the difference in multidimensional S/SDOH deprivation from other sociodemographic characteristics of older Black adults, such as different age groups or marital status. Third, differences in age, sex, education, and residential deprivation may limit generalizability to all Black adults ≥ 45 who are cognitively normal, and that our St. Louis–area sampling frame further constrains geographic external validity.

The study provides evidence that S/SDOH affects ADRD risk in an ethnoracial minority. We also show that a multidimensional poverty approach is more effective in showing this association between S/SDOH and ADRD risk than other indicators of SES, such as the ADI or the SVI, primarily used in the aging literature. This study advocates for ambitious, systemic socioeconomic interventions to reduce racial and ethnic structural injustice, resulting in an unfair allocation of S/SDOH during the life course and in brain health disparities. It offers insight into a new and promising measure of S/SDOH that allows for an in-depth understanding of multiple factors that influence ADRD risk during the life course.

## Supplementary Material

Supplement 1

Supplement 3

Supplement 2

Supplement 4

The online version contains supplementary material available at https://doi.org/10.1007/s40615-025-02730-0.

## Figures and Tables

**Table 1:** Sample demographic characteristics by dementia status

	CN^a^ (*N* = 207)	PCI^b^ (*N* = 105)	*P*-value	CN^c^ (*N* = 265)	PCP^d^ (*N* = 47)	*P*-value^e^
Sex						
Male	44 (21.3%)	26 (24.8%)	0.577	51 (19.2%)	19 (40.4%)	0.00254
Female	163 (78.7%)	79 (75.2%)		214 (80.8%)	28 (59.6%)	
Age						
Mean (SD)	63.4 (9.57)	66.7 (10.2)	0.00578	63.3 (9.44)	71.4 (9.75)	< 0.001
Median [Min, Max]	64.4 [45.0, 92.9]	66.8 [45.7, 91.9]		64.1 [45.0, 89.5]	71.8 [48.4, 92.9]	
Age group						
< 65	49 (23.7%)	15 (14.3%)	0.0732	62 (23.4%)	2 (4.3%)	0.00513
>=65	158 (76.3%)	90 (85.7%)		203 (76.6%)	45 (95.7%)	
Education (in years)						
Mean (SD)	15.3 (2.60)	14.3 (2.86)	0.00449	15.2 (2.64)	14.0 (2.99)	0.0122
Median [Min, Max]	16.0 [9.00, 29.0]	14.0 [8.00, 24.0]		16.0 [9.00, 29.0]	12.0 [8.00, 24.0]	
Marital status						
Married	50 (24.2%)	20 (19.0%)	0.0252	61 (23.0%)	9 (19.1%)	0.0955
Widowed	21 (10.1%)	14 (13.3%)		30 (11.3%)	5 (10.6%)	
Divorced	71 (34.3%)	30 (28.6%)		88 (33.2%)	13 (27.7%)	
Separated	4 (1.9%)	11 (10.5%)		9 (3.4%)	6 (12.8%)	
Never married (or marriage was annulled)	56 (27.1%)	28 (26.7%)		70 (26.4%)	14 (29.8%)	
Living as married/domestic partner	5 (2.4%)	2 (1.9%)		7 (2.6%)	0 (0%)	
Area Deprivation Index						
Mean (SD)	73.3 (22.7)	78.5 (20.8)	0.0456	74.3 (22.5)	79.5 (19.6)	0.105
Median [Min, Max]	80.0 [10.0, 100]	82.0 [20.0, 100]		81.0 [10.0, 100]	82.0 [21.0, 100]	
Social Vulnerability Index						
Mean (SD)	2.12 (2.08)	2.77 (2.47)	0.0207	2.23 (2.15)	2.96 (2.60)	0.0743
Median [Min, Max]	2.00 [0, 7.00]	2.00 [0, 7.00]		2.00 [0, 7.00]	2.00 [0, 7.00]	

The Montreal Cognitive Assessment (MoCA) and the preclinical Alzheimer’s cognitive composite (PACC) used to assess the cognitive status of participants.

a Cognitively normal defined by MoCA score above 23;

b Possible cognitive impairment defined by MoCA score equal or below 23;

c Cognitively normal defined by PACC score above − 1 standard deviation from the mean;

d poor cognitive performance defined by PACC score below − 1 standard deviation from the mean;

e: p value is calculated by t test for continuous variable and chi-square for categorial variable

**Table 2:** Logistic regression results of multidimensional poverty on dementia risk adjusted for age, gender, and marital status

	PCI^a^			PCI^a^			9CP^b^		PCP^b^			
			
	Unadjusted model^c^			Adjusted model			Unadjusted model^c^			Adjusted model		
			
*Predictors*	*OR*	*CI*	*p value*	*OR*	*CI*	*p value*	*OR*	*CI*	*p value*	*OR*	*CI*	*p value*
(Intercept)	0.45	0.34–0.58	<0.001	0.39	0.12–1.22	0.111	0.16	0.11–0.22	< 0.001	0.40	0.09–1.64	0.216
S/SDOH burden^d^	6.69	1.43–31.70	0.016	11.48	2.08–65.00	0.005	3.84	0.53–23.63	0.161	13.84	1.27–152.07	0.030
Age (ref: 65 and below)				1.90	1.14–3.22	0.015				6.49	2.84–16.85	<0.001
Sex (ref: male)				0.90	0.50–1.63	0.712				0.29	0.14–0.62	0.001
Marital status				0.82	0.45–1.45	0.503				0.62	0.25–1.42	0.280
(ref: Single)												
Observations	312			312			312			312		
R^2^ Tjur^e^	0.020			0.041			0.006			0.110		

*OR* odds ratio; *CI* confidence interval

The Montreal Cognitive Assessment (MoCA) and the preclinical Alzheimer’s cognitive composite (PACC) used to assess the cognitive status of participants

a Possible cognitive impairment (PCI) defined by MoCA score equal or below 23

b Poor cognitive performance (PCP) defined by PACC score below − 1 standard deviation from the mean

c The unadjusted model includes no covariates

d S/SDOH burden is obtained by dividing the weighted S/SDOH indicators on which an individual was deprived by the total weighted indicators among individuals deprived on 11 S/SDOH indicators or more

e R2 Tjur: pseudo R square

## Data Availability

Data can be made available up request.
