# Supplementary material for: Association Between Structural and Social Determinants of Health and Cognitive Functioning Among African Americans: the ARCHES Cohort
Source: J Racial Ethn Health Disparities. Author manuscript; Available in PMC 2026 Feb 6. (PMC12875771; doi:10.1007/s40615-025-02730-0)
Supplement: Supplement 3 [file NIHMS2126819-supplement-Supplement_3.docx]

**Supplementary file: Structural and Social Determinants of Health Composite Index (S/SDOH-CI) based on the three dimensions of the National Institute of Aging (NIA) Health Disparities Research Framework (HDRF)**

The S/SDOH-CI is built on the multidimensional poverty approach. It is composed of 37 indicators of deprivation across ten factors within the behavioral, environmental and social dimensions of the NIA-HDRF. All indicators contribute together but in different proportions to AD/ADRD risk. Detailed descriptions of the dimensions, factors, indicators, and their respective cut-off points for defining deprivation of resources to support health are described in the present note.

Environmental dimension

Environmental factors included neighborhood social cohesion and exchange alongside social and physical disorder. The size, diversity, and quality of one’s social network and the broader sense of community connectedness have demonstrable health impacts [1]. Conversely, visible signs of social decline and neighborhood deterioration are linked to greater social isolation and poorer physical health in older adults [2, 3]. Deprivation cut-offs were defined as one standard deviation below the mean on the social cohesion subscale and one above the mean on the physical disorder subscale.

Among socioeconomic factors, quality of education is an established and significant determinant of health that impacts one’s type of employment, influences earnings and quality healthcare, reduces financial problems at older age, and builds the capacity to communicate [4, 5]. Two indicators of quality education included education level and reading skill. Participants were considered deprived if they did not attend college and scored 1 SD below the mean on the Wide Range Achievement Test (WRAT) reading subtest [6].

Household living standards were assessed using four indicators: perceived living conditions, transportation, communication, and an asset index. The deprivation cut-off was defined as not owning a home and residing in overcrowded conditions (more than two persons per room), lacking access to a car, or not having internet connectivity. The asset index comprised commonly owned items in U.S. households, including televisions, computers, phones, refrigerators, dishwashers, bicycles, washing machines, motorcycles, and vehicles. This index was constructed using polychoric principal component analysis, with the first principal component capturing the largest share of variance, supporting a strong one-factor structure [7]. A score below one SD below the mean was used as the deprivation cut-off.

Barriers to accessing quality healthcare were measured using a 17-item questionnaire adapted from the Barriers to Care Scale (BACS) [8]. It consisted of nine components: emotional barriers, executive functioning, healthcare navigation, provider attitudes, communication, sensory challenges, socioeconomic status, support from family and friends, and waiting room experiences. Participants were classified as deprived in a given component if they responded “yes” to a predefined number of items indicating difficulty in that domain.

Sociocultural dimension

The first factor within the sociocultural dimension, religiosity, was measured using the Duke University Religion Index, a five-item instrument assessing religious service attendance, private religious practices, and intrinsic religiosity across three subscales [9]. Religiosity has been linked to reduced anxiety and depression, and to enhanced life satisfaction, meaning in life, resilience, social connections, and psychological well-being [10-13]. Willis et al. (2019) further identified a significant positive association between religious doubt and depressive symptoms among Black, but not White, participants [14]. The cut-off was defined as scoring one standard deviation below the mean on each subscale.

The social factor within the sociocultural dimension comprised two indicators. The first, a six-item hardship scale, measured the frequency with which families experienced difficulty affording basic needs such as stable housing and food. Participants were classified as deprived if they reported difficulty accessing any of these needs. The second indicator captured exposure to life stressors, including health issues, financial strain, housing instability, and burdensome caregiving responsibilities. Black American adults are disproportionately exposed to chronic stress due to socioeconomic disadvantage and political marginalization, contributing to a higher allostatic load—the cumulative physiological toll of stress—compared to non-Hispanic Whites, irrespective of income level  [15, 16]. Life stressors were assessed using the 8-item Ongoing Chronic Stressors scale [17], which evaluates the presence and perceived severity of common stressors over the past year or longer, with responses ranging from “No Didn’t happen” to “Yes, Very Upsetting” [17]. The deprivation cut-off was set at one SD above the mean.

Personality and life satisfaction were the two indicators representing the psychological factor within the sociocultural dimension. Personality was assessed using the 50-item International Personality Item Pool (IPIP), which measures the five major traits: extraversion, agreeableness, openness, conscientiousness, and neuroticism [18]. Among these, neuroticism and conscientiousness have been particularly linked to ADRD risk [19-22]. The deprivation cut-off was set at one SD below the mean on extraversion, agreeableness, openness, and conscientiousness and one SD above the mean on neuroticism.

The indicator for life satisfaction was measured using the five-item Satisfaction with Life Scale, which assesses overall happiness through statements rated on a seven-point scale ranging from "strongly agree" to "strongly disagree" [23]. As a standard indicator of well-being, life satisfaction is commonly used to evaluate healthy aging [24] and has been linked to dementia risk among older adults [25, 26]. Higher scores reflect greater life satisfaction, with the deprivation cut-off defined as a score of one SD below the mean.

Behavioral dimension

The third and final dimension of the HDRF, the behavioral dimension, comprised three factors. The first factor assessed coping capability and vigilance. Coping with psychosocial stressors used the 12-item John Henryism Scale, developed specifically for Black Americans to reflect their unique social and cultural contexts [27]. John Henryism refers to sustained high-effort coping in response to chronic stressors, often rooted in structural racism and socioeconomic disadvantage [28, 29]. While adaptive in some contexts, high levels of John Henryism have been associated with adverse physical health outcomes [27, 30]. A score exceeding one SD above the mean indicated high-effortful coping and was used as the deprivation cut-off.

Vigilance was assessed using the Heightened Vigilance Scale, a six-item instrument that gauges the cognitive and behavioral efforts individuals make to guard against potential discrimination by continuously monitoring and adjusting their behavior [31, 32]. This form of vigilance, regarded as a passive coping strategy, has been linked to elevated stress [33] and adverse health outcomes, including hypertension [34], sleep disturbances [32], and adverse cardiovascular effects [31]. A score of one SD above the mean served as the deprivation cut-off.

The second factor, psychological risk and resilience, was evaluated using the 9-item Perceived Everyday Discrimination Scale, which measures the frequency of chronic and routine discrimination in daily situations [35]. Existing research on the association between discrimination and cognitive health (based on biomarkers or cognitive testing) has yielded mixed findings [36, 37]. The deprivation cut-off was a score of one SD above the mean.

The third and final factor focused on health behaviors known to affect cognitive outcomes. Stress often triggers passive coping mechanisms deleterious to health, such as consuming high-fat, calorie-dense comfort foods, smoking, and using alcohol or illicit drugs [38, 39]. We examined seven types of behavioral patterns (e.g., currently smoking, alcohol use, substance abuse, physical inactivity, and food insecurity) that are potentially detrimental to health, establishing specific deprivation cut-off for each (Supplement Table 1).

References

1. Kim, D. and I. Kawachi, *A multilevel analysis of key forms of community-and individual-level social capital as predictors of self-rated health in the United States.* Journal of Urban Health, 2006. **83**: p. 813-826.

2. Krause, N., *Neighborhood deterioration and self-rated health in later life.* Psychology and aging, 1996. **11**(2): p. 342.

3. Krause, N., *Neighborhood deterioration and social isolation in later life.* The International Journal of Aging and Human Development, 1993. **36**(1): p. 9-38.

4. Mirowsky, J., *Education, social status, and health*. 2017: Routledge.

5. Schulz, J.H., *The economics of aging*. 2000: Bloomsbury Publishing USA.

6. Wilkinson, G.S. and G.J. Robertson, *Wide range achievement test 4 (WRAT4).* Lutz, FL: Psychological Assessment Resources, 2006.

7. Kolenikov, S. and G. Angeles, *Socioeconomic status measurement with discrete proxy variables: is principal component analysis: a reliable answer?* Review of Income and Wealth, 2009. **55**(1): p. 128-165.

8. Heckman, T.G., et al., *Barriers to care among persons living with HIV/AIDS in urban and rural areas.* AIDS care, 1998. **10**(3): p. 365-375.

9. Koenig, H.G. and A. Büssing, *The Duke University Religion Index (DUREL): a five-item measure for use in epidemological studies.* Religions, 2010. **1**(1): p. 78-85.

10. Coelho-Júnior, H.J., et al., *Religiosity/spirituality and mental health in older adults: A systematic review and meta-analysis of observational studies.* Frontiers in Medicine, 2022. **9**: p. 877213.

11. Manning, L.K. and A. Miles, *Examining the effects of religious attendance on resilience for older adults.* Journal of religion and health, 2018. **57**: p. 191-208.

12. McGowan, J.C., et al., *Religiousness and psychological distress in Jewish and Christian older adults.* Clinical Gerontologist, 2016. **39**(5): p. 489-507.

13. Cruz, M., et al., *The association of public and private religious involvement with severity of depression and hopelessness in older adults treated for major depression.* The American journal of geriatric psychiatry, 2009. **17**(6): p. 503-507.

14. Willis, K.D., T. Nelson, and O. Moreno, *Death anxiety, religious doubt, and depressive symptoms across race in older adults.* International Journal of Environmental Research and Public Health, 2019. **16**(19): p. 3645.

15. McEwen, B.S., *Protective and damaging effects of stress mediators.* New England journal of medicine, 1998. **338**(3): p. 171-179.

16. Geronimus, A.T., et al., *“Weathering” and age patterns of allostatic load scores among blacks and whites in the United States.* American journal of public health, 2006. **96**(5): p. 826-833.

17. Troxel, W.M., et al., *Chronic stress burden, discrimination, and subclinical carotid artery disease in African American and Caucasian women.* Health Psychology, 2003. **22**(3): p. 300.

18. Donnellan, M.B., et al., *The mini-IPIP scales: tiny-yet-effective measures of the Big Five factors of personality.* Psychological assessment, 2006. **18**(2): p. 192.

19. Duchek, J.M., et al., *The Power of Personality in Discriminating Between Healthy Aging and Early-Stage Alzheimer's Disease.* The Journals of Gerontology: Series B, 2007. **62**(6): p. P353-P361.

20. Duchek, J.M., et al., *The relation between personality and biomarkers in sensitivity and conversion to Alzheimer-type dementia.* Journal of the International Neuropsychological Society, 2020. **26**(6): p. 596-606.

21. Aschenbrenner, A.J., et al., *Relationships between big‐five personality factors and Alzheimer's disease pathology in autosomal dominant Alzheimer's disease.* Alzheimer's & Dementia: Diagnosis, Assessment & Disease Monitoring, 2020. **12**(1): p. e12038.

22. Terracciano, A. and A.R. Sutin, *Personality and Alzheimer’s disease: An integrative review.* Personality Disorders: Theory, Research, and Treatment, 2019. **10**(1): p. 4.

23. Diener, E.D., et al., *The satisfaction with life scale.* Journal of Personality Assessment, 1985. **49**(1): p. 71-75.

24. Torregrosa-Ruiz, M., et al., *A successful aging model based on personal resources, self-care, and life satisfaction.* The Journal of Psychology, 2021. **155**(7): p. 606-623.

25. Peitsch, L., et al., *General life satisfaction predicts dementia in community living older adults: a prospective cohort study.* International Psychogeriatrics, 2016. **28**(7): p. 1101-1109.

26. Zhu, X., et al., *Satisfaction with life and risk of dementia: Findings from the Korean Longitudinal Study of Aging.* The Journals of Gerontology: Series B, 2022. **77**(10): p. 1831-1840.

27. James, S.A., S.A. Hartnett, and W.D. Kalsbeek, *John Henryism and blood pressure differences among black men.* Journal of behavioral medicine, 1983. **6**(3): p. 259-278.

28. Mitchell, R., *Lifetime Racism and John Henryism on Cognition and Cardiovascular Health in Black Men.* 2022.

29. James, S.A., *John Henryism, Structural Racism, and Cardiovascular Health Risks in Black Americans*, in *Racism: Science & Tools for the Public Health Professional*. 2019.

30. James, S.A., et al., *Socioeconomic status, John Henryism, and hypertension in blacks and whites.* American journal of epidemiology, 1987. **126**(4): p. 664-673.

31. Clark, R., R.A. Benkert, and J.M. Flack, *Large Arterial Elasticity Varies as a Function of Gender and Racism-Related Vigilance in Black Youth.* Journal of Adolescent Health, 2006. **39**(4): p. 562-569.

32. Hicken, M.T., et al., *“Every shut eye, ain’t sleep”: The role of racism-related vigilance in racial/ethnic disparities in sleep difficulty.* Race and social problems, 2013. **5**: p. 100-112.

33. Himmelstein, M.S., et al., *Vigilance in the discrimination-stress model for Black Americans.* Psychol Health, 2015. **30**(3): p. 253-67.

34. Smith, T.W., J.M. Ruiz, and B.N. Uchino, *Vigilance, active coping, and cardiovascular reactivity during social interaction in young men.* Health Psychology, 2000. **19**(4): p. 382-392.

35. Williams, D.R., et al., *Racial differences in physical and mental health: Socio-economic status, stress and discrimination.* Journal of Health Psychology, 1997. **2**(3): p. 335-351.

36. Chen, R., et al., *Associations of Major Lifetime and Everyday Discrimination with Cognitive Function among Middle-Aged and Older Adults.* Ethnicity & Disease, 2024. **34**(3): p. 137-144.

37. Lawrence, J.A., et al., *A systematic review and meta-analysis of the Everyday Discrimination Scale and biomarker outcomes.* Psychoneuroendocrinology, 2022. **142**: p. 105772.

38. Hoggard, L.S., et al., *The role of emotional eating in the links between racial discrimination and physical and mental health.* Journal of Behavioral M

edicine, 2019. **42**(6): p. 1091-1103.

39. Rodriquez, E.J., et al., *Coping with chronic stress by unhealthy behaviors: a re-evaluation among older adults by race/ethnicity.* Journal of aging and health, 2017. **29**(5): p. 805-825.
