# Supplementary material for: Association Between Structural and Social Determinants of Health and Cognitive Functioning Among African Americans: the ARCHES Cohort
Source: J Racial Ethn Health Disparities. Author manuscript; Available in PMC 2026 Feb 6. (PMC12875771; doi:10.1007/s40615-025-02730-0)
Supplement: Supplement 4 [file NIHMS2126819-supplement-Supplement_4.docx]

**Supplementary material**

Supplementary Table 1. Dimensions, factors, and indicators of deprivation

Supplementary Table 2. Proportion of participants deprived in each dimension by PCI or PCP, gender, and age group

Supplementary Table 3 Spearman rank correlations between indicators of deprivation

Supplementary Table 4 Multidimensional poverty measures for Black adults by cognitive status, gender and age

Supplementary Table 5 Indicator’s contributions to deprivation of S/SDOH by cognitive status, sex, and age groups

Supplementary Table 6a. Logistic regression results of area deprivation index on dementia risk-adjusted for age, gender, and marital status.

Supplementary Table 6b. Logistic regression results of Social Vulnerability Index on Dementia risk adjusted for age, gender, and marital status.

Supplementary Table 1. Dimensions, factors and indicators of deprivation

| **Dimensions factors and indicators** | **Indicators definition** | **Deprivation cutoff (Deprived if…)** |  |
| --- | --- | --- | --- |
| **Environmental dimension** |  |  |  |
| *Geographical and political factor (urban/suburban)* |  |  |  |
| *1. Neighborhood social cohesion* | Social cohesion. The 8-item neighborhood disorder/social cohesion survey examines social cohesion and physical disorder at the neighborhood level (~ 1-mile) via subjective and objective characteristics of the residential environment | The score on questions 2, 4, 6 and 8 for social cohesion is one standard deviation (SD) above the mean. |  |
| *2. Neighborhood disorder* |  | The score on questions 1, 3, 5 and 7 for neighborhood disorder is one standard deviation (SD) above the mean. |  |
| *Socioeconomic factor (education)* |  |  |  |
| *3.Number of years of education* | How many years of education do you have? | Less than 12 years of education. |  |
|  |  |  |  |
| *4. Quality of the education received* | Reading skill as a proxy for education quality is assessed by the Wide Range Achievement Test (WRAT) reading subtest. | The score on the WRAT is one standard deviation (SD) below the mean. |  |
| *Socioeconomic factor (Household living standards)* |  |  |  |
| *5 Living conditions* | Living condition | Participants do not own a home and live in a crowded place with more than two people per room. |  |
| *6 Transportation* | Transportation | Participants do not own a car. |  |
| *7 Communication* | Communication | Participants do not own cell phone or home internet. |  |
| *8 Assets* | Asset index is assessed using a composite index that includes a list of common property possessed by a US household (houses, cars or other vehicles, savings) | The score on the asset index is one standard deviation (SD) below the mean. |  |
| *Barriers to accessing quality healthcare* |  |  |  |
| *9 Emotional* | Barriers to accessing quality healthcare were measured using a 17-item questionnaire adapted from the Barriers to Care Scale (BACS) | Participants responded "yes" to the question assessing emotional barriers. |  |
| *10 Executive function* |  | Participants responded ""yes" to either of the two questions assessing executive function barriers. |  |
| *11 Healthcare navigation* |  | Participants respond "yes" to at least one of three questions assessing healthcare navigation barriers. |  |
| *12 Provider attitude* |  | Participants respond "yes" to at least one of four questions assessing provider attitude barriers. |  |
| *13 Patient provider communication* |  | Participants respond "yes" to either of the two questions assessing communication barriers. |  |
| 14 Sensory | Sensory barriers | Participants respond "yes" to the question assessing sensory barriers. |  |
| 15 Socio-economic status | Socio-economic status barriers | Participants respond "yes" to either of the two questions assessing SES barriers. |  |
| 16 Family and friends support | Family and friends support barriers | Participants respond "yes" to the question assessing family and friends’ barriers. |  |
| 17 Waiting room | Waiting room barriers | Participants respond "yes" to the question assessing waiting room barriers. |  |
| **Sociocultural dimension** |  |  |  |
| *Cultural factor Religiosity* |  |  |  |
| *18 Attending religious services* | Frequency of attending religious activities measured by the Duke University Religion Index | Participants respond "never" or "once a year or less" to the question of attending church or other religious meetings. |  |
| 19 Private religious activities | Frequency of attending religious activities outside church measured by the Duke University Religion Index | Participants respond "rarely" or "never" or "a few times a month" to the question of spending time in private religious activities, such as prayers meditation or bible study. |  |
| 20 Intrinsic religiosity | Intrinsic religiosity measured by the Duke University Religion Index | Participants rate “how true” each of three statements about intrinsic religious value are. Values range from 1 “not true” to 5 “definitely true”. One SD below the mean score on the three statements is considered as deprived. |  |
| *Social factor* |  |  |  |
| *21 Hardship* | The Hardship scale consists of six questions assessing the frequency of a family having difficulty to afford basic needs. | Participants respond somewhat hard to pay for basic need, or not able to pay rent or mortgage or not having a steady place to stay, or 2 or greater on the number of places one has lived or “1 to 4 times” or “more than four times” having difficulty to buy food, or “1 to 4 times” or “more than 4 times” did not have money to get enough food when food was running out. |  |
| *22 Experiencing life stressors* | The Ongoing Chronic Stressors Scale consists of eight items asking about experiencing life stressors in the past one year. | One SD above the mean is considered as “deprived”. |  |
| *Psychological factor* |  |  |  |
| *Personality traits*  *23.Extraversion*  *24 Agreeableness*  *25 Openness*  *26* Conscientiousness  27 Neuroticism | The 50-item International Personality Item Pool is composed of five scores: extraversion, openness, agreeableness, conscientiousness, neuroticism. | One SD below the mean score on each of these scores is considered as deprived on that personality trait, except for neuroticism for which one SD above the mean is considered as deprived. |  |
| *28. Life satisfaction* | The 5-item Satisfaction with Life Scale encompasses statements about satisfaction with life on a seven-point scale (from 1 strongly disagree to 7 strongly agree). | One SD below the mean score is considered as deprived. |  |
| **Behavioral dimension** |  |  |  |
| *Coping factors* |  |  |  |
| *29. Coping capability* | The 12-item John Henryism Active Coping Scale assesses the individual’s ability to cope with psychosocial stressors and was designed for Black Americans. Content includes commitment to hard work, mental and physical vigor, and self-determination to achieve goals. Each item is evaluated on a how true/false five-point scale ranging from 1 “completely false” to 5 “completely true”. | One SD above the mean score is considered as "overcoping" , and in turns is considered as "deprived" . |  |
| *30 Vigilance against discrimination* | The Heightened Vigilance Scale established for the Detroit Area Study at the University of Michigan—Institute for Social Research is a 6-item questionnaire with a 5-point Likert scale ranging from 1 “never” to 5 “very often”. The higher the score, the higher the level of vigilance. | One SD above the mean score is considered as "deprived". |  |
| *Psychosocial Risk/Resilience* |  |  |  |
| *31. Perceived everyday discrimination* | The Perceived Everyday Discrimination Scale is a 9-item scale assessing frequency of everyday life discrimination ranging from 6 “very often” to 1 “never”. | One SD above the mean score is considered "deprived". |  |
| *Health Behaviors (Healthy behavior and risk factors):* |  |  |  |
| *32. Physical Activity* | Physical activities measured by International Physical Activity Questionnaire Measure of volume of activity in the past week is computed by weighting each type of activity by its energy requirements defined in METS (METs are multiples of the resting metabolic rate) to yield a score in MET minutes. | -1SD of MET minutes is considered as "deprived". |  |
| 33. Sitting | Time spent sitting measured by International Physical Activity Questionnaire | Time spent sitting 1SD above the mean in the past week is considered as “deprived”. |  |
| *34. Smoking* | Currently smoking | Smoking in the past 30 days is considered as “deprived”. |  |
| *35. Alcohol consumption* | Phenx Alcohol | Having more than 1 drink per day/ more than 7 drinks per week is considered as deprived |  |
| *36. Substances use* | Phenx Substances | Using any type of drug is considered as deprived |  |
| *37. Household Food Insecurity* | The Household Food insecurity and Access Prevalence (HFIAP) scale categorizes households into four levels of household food insecurity (access): food secure, and mild, moderately and severely food insecure. | Mild food insecurity and above is considered as deprived. |  |

Supplementary Table 2a Proportion of participants deprived in each dimension by PCI, gender, and age group

|  | Overall | | | Female | | | | | Male | | | | Below 65 | | | | 65 and above | | | |
| --- | --- | --- | --- | --- | --- | --- | --- | --- | --- | --- | --- | --- | --- | --- | --- | --- | --- | --- | --- | --- |
|  | CN^a^ | PCI^b^ | P-value | | CN^a^ | PCI^b^ | P-value | CN^a^ | | PCI^b^ | P-value | CN^a^ | | PCI^b^ | P-value | CN^a^ | | PCI^b^ | P-value |  |
|  | (N=207) | (N=105) |  | | (N=163) | (N=79) |  | (N=44) | | (N=26) |  | (N=107) | | (N=44) |  | (N=100) | | (N=61) |  |  |
| Neighborhood cohesiveness | 41 (19.8%) | 26 (24.8%) | 0.389 | | 32 (19.6%) | 19 (24.1%) | 0.534 | 9 (20.5%) | | 7 (26.9%) | 0.743 | 23 (21.5%) | | 10 (22.7%) | 1 | 18 (18.0%) | | 16 (26.2%) | 0.297 |  |
| Neighborhood disorder | 29 (14.0%) | 26 (24.8%) | 0.028 | | 23 (14.1%) | 19 (24.1%) | 0.083 | 6 (13.6%) | | 7 (26.9%) | 0.288 | 18 (16.8%) | | 11 (25.0%) | 0.351 | 11 (11.0%) | | 15 (24.6%) | 0.0401 |  |
| Years of education | 46 (22.2%) | 42 (40.0%) | 0.00155 | | 31 (19.0%) | 24 (30.4%) | 0.0697 | 15 (34.1%) | | 18 (69.2%) | 0.00938 | 30 (28.0%) | | 24 (54.5%) | 0.00372 | 16 (16.0%) | | 18 (29.5%) | 0.066 |  |
| Reading skills | 14 (6.8%) | 37 (35.2%) | <0.001 | | 12 (7.4%) | 24 (30.4%) | <0.001 | 2 (4.5%) | | 13 (50.0%) | <0.001 | 7 (6.5%) | | 22 (50.0%) | <0.001 | 7 (7.0%) | | 15 (24.6%) | 0.00355 |  |
| Living conditions | 31 (15.0%) | 20 (19.0%) | 0.449 | | 22 (13.5%) | 18 (22.8%) | 0.101 | 9 (20.5%) | | 2 (7.7%) | 0.281 | 22 (20.6%) | | 13 (29.5%) | 0.329 | 9 (9.0%) | | 7 (11.5%) | 0.812 |  |
| Transportation | 33 (15.9%) | 21 (20.0%) | 0.461 | | 22 (13.5%) | 12 (15.2%) | 0.874 | 11 (25.0%) | | 9 (34.6%) | 0.557 | 19 (17.8%) | | 15 (34.1%) | 0.0489 | 14 (14.0%) | | 6 (9.8%) | 0.596 |  |
| Communication | 5 (2.4%) | 8 (7.6%) | 0.061 | | 4 (2.5%) | 3 (3.8%) | 0.86 | 1 (2.3%) | | 5 (19.2%) | 0.0447 | 4 (3.7%) | | 6 (13.6%) | 0.0625 | 1 (1.0%) | | 2 (3.3%) | 0.662 |  |
| Assets | 37 (17.9%) | 16 (15.2%) | 0.67 | | 27 (16.6%) | 13 (16.5%) | 1 | 10 (22.7%) | | 3 (11.5%) | 0.398 | 26 (24.3%) | | 9 (20.5%) | 0.767 | 11 (11.0%) | | 7 (11.5%) | 1 |  |
| Emotional barriers | 6 (2.9%) | 7 (6.7%) | 0.203 | | 4 (2.5%) | 3 (3.8%) | 0.86 | 2 (4.5%) | | 4 (15.4%) | 0.261 | 6 (5.6%) | | 5 (11.4%) | 0.372 | 0 (0%) | | 2 (3.3%) | 0.276 |  |
| Executive function barriers | 25 (12.1%) | 11 (10.5%) | 0.817 | | 17 (10.4%) | 6 (7.6%) | 0.637 | 8 (18.2%) | | 5 (19.2%) | 1 | 19 (17.8%) | | 6 (13.6%) | 0.705 | 6 (6.0%) | | 5 (8.2%) | 0.831 |  |
| Healthcare navigation barriers | 39 (18.8%) | 30 (28.6%) | 0.0699 | | 26 (16.0%) | 18 (22.8%) | 0.265 | 13 (29.5%) | | 12 (46.2%) | 0.253 | 23 (21.5%) | | 18 (40.9%) | 0.0253 | 16 (16.0%) | | 12 (19.7%) | 0.702 |  |
| Provider attitudes barriers | 27 (13.0%) | 21 (20.0%) | 0.149 | | 17 (10.4%) | 13 (16.5%) | 0.26 | 10 (22.7%) | | 8 (30.8%) | 0.645 | 20 (18.7%) | | 10 (22.7%) | 0.734 | 7 (7.0%) | | 11 (18.0%) | 0.0578 |  |
| Patient-provider communication barriers | 17 (8.2%) | 6 (5.7%) | 0.57 | | 13 (8.0%) | 4 (5.1%) | 0.573 | 4 (9.1%) | | 2 (7.7%) | 1 | 11 (10.3%) | | 4 (9.1%) | 1 | 6 (6.0%) | | 2 (3.3%) | 0.691 |  |
| Sensory barriers | 3 (1.4%) | 1 (1.0%) | 1 | | 1 (0.6%) | 0 (0%) | 1 | 2 (4.5%) | | 1 (3.8%) | 1 | 2 (1.9%) | | 1 (2.3%) | 1 | 1 (1.0%) | | 0 (0%) | 1 |  |
| SES barriers | 23 (11.1%) | 17 (16.2%) | 0.276 | | 19 (11.7%) | 10 (12.7%) | 0.989 | 4 (9.1%) | | 7 (26.9%) | 0.101 | 17 (15.9%) | | 11 (25.0%) | 0.281 | 6 (6.0%) | | 6 (9.8%) | 0.555 |  |
| Support barriers | 31 (15.0%) | 19 (18.1%) | 0.585 | | 22 (13.5%) | 14 (17.7%) | 0.501 | 9 (20.5%) | | 5 (19.2%) | 1 | 17 (15.9%) | | 11 (25.0%) | 0.281 | 14 (14.0%) | | 8 (13.1%) | 1 |  |
| Waiting room barriers | 8 (3.9%) | 9 (8.6%) | 0.142 | | 6 (3.7%) | 6 (7.6%) | 0.318 | 2 (4.5%) | | 3 (11.5%) | 0.537 | 4 (3.7%) | | 4 (9.1%) | 0.35 | 4 (4.0%) | | 5 (8.2%) | 0.441 |  |
| Attending religious services | 47 (22.7%) | 15 (14.3%) | 0.107 | | 26 (16.0%) | 9 (11.4%) | 0.453 | 21 (47.7%) | | 6 (23.1%) | 0.0729 | 35 (32.7%) | | 8 (18.2%) | 0.11 | 12 (12.0%) | | 7 (11.5%) | 1 |  |
| Private religious activities | 61 (29.5%) | 24 (22.9%) | 0.269 | | 38 (23.3%) | 19 (24.1%) | 1 | 23 (52.3%) | | 5 (19.2%) | 0.0134 | 38 (35.5%) | | 12 (27.3%) | 0.431 | 23 (23.0%) | | 12 (19.7%) | 0.764 |  |
| Intrinsic religiosity | 23 (11.1%) | 15 (14.3%) | 0.531 | | 11 (6.7%) | 8 (10.1%) | 0.508 | 12 (27.3%) | | 7 (26.9%) | 1 | 12 (11.2%) | | 9 (20.5%) | 0.218 | 11 (11.0%) | | 6 (9.8%) | 1 |  |
| Hardship | 36 (17.4%) | 15 (14.3%) | 0.59 | | 27 (16.6%) | 9 (11.4%) | 0.386 | 9 (20.5%) | | 6 (23.1%) | 1 | 25 (23.4%) | | 10 (22.7%) | 1 | 11 (11.0%) | | 5 (8.2%) | 0.76 |  |
| Life stressors | 120 (58.0%) | 74 (70.5%) | 0.0425 | | 91 (55.8%) | 52 (65.8%) | 0.179 | 29 (65.9%) | | 22 (84.6%) | 0.155 | 74 (69.2%) | | 33 (75.0%) | 0.603 | 46 (46.0%) | | 41 (67.2%) | 0.014 |  |
| Extraversion | 25 (12.1%) | 23 (21.9%) | 0.0351 | | 20 (12.3%) | 16 (20.3%) | 0.149 | 5 (11.4%) | | 7 (26.9%) | 0.18 | 18 (16.8%) | | 13 (29.5%) | 0.124 | 7 (7.0%) | | 10 (16.4%) | 0.106 |  |
| Agreeableness | 29 (14.0%) | 20 (19.0%) | 0.322 | | 20 (12.3%) | 11 (13.9%) | 0.876 | 9 (20.5%) | | 9 (34.6%) | 0.304 | 17 (15.9%) | | 12 (27.3%) | 0.166 | 12 (12.0%) | | 8 (13.1%) | 1 |  |
| Openness | 23 (11.1%) | 27 (25.7%) | 0.00158 | | 19 (11.7%) | 24 (30.4%) | <0.001 | 4 (9.1%) | | 3 (11.5%) | 1 | 10 (9.3%) | | 10 (22.7%) | 0.0524 | 13 (13.0%) | | 17 (27.9%) | 0.0322 |  |
| Conscientiousness | 27 (13.0%) | 20 (19.0%) | 0.217 | | 20 (12.3%) | 13 (16.5%) | 0.49 | 7 (15.9%) | | 7 (26.9%) | 0.421 | 19 (17.8%) | | 9 (20.5%) | 0.875 | 8 (8.0%) | | 11 (18.0%) | 0.0964 |  |
| Neuroticism | 28 (13.5%) | 25 (23.8%) | 0.0335 | | 20 (12.3%) | 16 (20.3%) | 0.149 | 8 (18.2%) | | 9 (34.6%) | 0.207 | 22 (20.6%) | | 13 (29.5%) | 0.329 | 6 (6.0%) | | 12 (19.7%) | 0.0158 |  |
| Life satisfaction | 42 (20.3%) | 16 (15.2%) | 0.352 | | 26 (16.0%) | 10 (12.7%) | 0.63 | 16 (36.4%) | | 6 (23.1%) | 0.373 | 28 (26.2%) | | 9 (20.5%) | 0.594 | 14 (14.0%) | | 7 (11.5%) | 0.826 |  |
| Coping capability | 28 (13.5%) | 12 (11.4%) | 0.73 | | 21 (12.9%) | 11 (13.9%) | 0.983 | 7 (15.9%) | | 1 (3.8%) | 0.253 | 12 (11.2%) | | 7 (15.9%) | 0.603 | 16 (16.0%) | | 5 (8.2%) | 0.236 |  |
| Vigilance | 35 (16.9%) | 19 (18.1%) | 0.918 | | 25 (15.3%) | 14 (17.7%) | 0.774 | 10 (22.7%) | | 5 (19.2%) | 0.966 | 27 (25.2%) | | 9 (20.5%) | 0.677 | 8 (8.0%) | | 10 (16.4%) | 0.167 |  |
| Everyday discrimination | 26 (12.6%) | 17 (16.2%) | 0.481 | | 15 (9.2%) | 11 (13.9%) | 0.373 | 11 (25.0%) | | 6 (23.1%) | 1 | 23 (21.5%) | | 10 (22.7%) | 1 | 3 (3.0%) | | 7 (11.5%) | 0.068 |  |
| Physical activity | 14 (6.8%) | 6 (5.7%) | 0.91 | | 10 (6.1%) | 5 (6.3%) | 1 | 4 (9.1%) | | 1 (3.8%) | 0.732 | 7 (6.5%) | | 1 (2.3%) | 0.506 | 7 (7.0%) | | 5 (8.2%) | 1 |  |
| Sitting | 13 (6.3%) | 5 (4.8%) | 0.774 | | 8 (4.9%) | 4 (5.1%) | 1 | 5 (11.4%) | | 1 (3.8%) | 0.52 | 9 (8.4%) | | 2 (4.5%) | 0.627 | 4 (4.0%) | | 3 (4.9%) | 1 |  |
| Smoking | 31 (15.0%) | 17 (16.2%) | 0.908 | | 20 (12.3%) | 8 (10.1%) | 0.784 | 11 (25.0%) | | 9 (34.6%) | 0.557 | 23 (21.5%) | | 13 (29.5%) | 0.398 | 8 (8.0%) | | 4 (6.6%) | 0.977 |  |
| Alcohol | 48 (23.2%) | 26 (24.8%) | 0.867 | | 34 (20.9%) | 16 (20.3%) | 1 | 14 (31.8%) | | 10 (38.5%) | 0.76 | 25 (23.4%) | | 15 (34.1%) | 0.248 | 23 (23.0%) | | 11 (18.0%) | 0.582 |  |
| Substance use | 103 (49.8%) | 47 (44.8%) | 0.475 | | 71 (43.6%) | 30 (38.0%) | 0.492 | 32 (72.7%) | | 17 (65.4%) | 0.706 | 57 (53.3%) | | 24 (54.5%) | 1 | 46 (46.0%) | | 23 (37.7%) | 0.386 |  |
| Food insecurity | 34 (16.4%) | 14 (13.3%) | 0.583 | | 26 (16.0%) | 7 (8.9%) | 0.191 | 8 (18.2%) | | 7 (26.9%) | 0.576 | 25 (23.4%) | | 11 (25.0%) | 0.997 | 9 (9.0%) | | 3 (4.9%) | 0.517 |  |

Notes: The Montreal Cognitive Assessment (MoCA) was used to assess the cognitive status of participants.

a CN: Cognitive normal

b Possible cognitive impairment (PCI) defined by MoCA score equal or below 23

Supplementary Table 2b. Proportion of participants deprived in each dimension by PCP, gender, and age group

|  | Overall | | | Female | | | Male | | | Below 65 | | | 65 and above | | |
| --- | --- | --- | --- | --- | --- | --- | --- | --- | --- | --- | --- | --- | --- | --- | --- |
|  | CN^a^ | PCP^b^ | P-value | CN^a^ | PCP^b^ | P-value | CN^a^ | PCP^b^ | P-value | CN^a^ | PCP^b^ | P-value | CN^a^ | PCP^b^ | P-value |
|  | (N=125) | (N=36) |  | (N=214) | (N=28) |  | (N=50) | (N=19) |  | (N=139) | (N=11) |  | (N=125) | (N=36) |  |
| Neighborhood cohesiveness | 23 (18.4%) | 11 (30.6%) | 0,179 | 41 (19.2%) | 10 (35.7%) | 0,0761 | 11 (22.0%) | 4 (21.1%) | 1 | 29 (20.9%) | 3 (27.3%) | 0,907 | 23 (18.4%) | 11 (30.6%) | 0,179 |
| Neighborhood disorder | 17 (13.6%) | 9 (25.0%) | 0,167 | 34 (15.9%) | 8 (28.6%) | 0,161 | 7 (14.0%) | 5 (26.3%) | 0,395 | 24 (17.3%) | 4 (36.4%) | 0,245 | 17 (13.6%) | 9 (25.0%) | 0,167 |
| Years of education | 18 (14.4%) | 16 (44.4%) | <0.001 | 43 (20.1%) | 12 (42.9%) | 0,0138 | 20 (40.0%) | 12 (63.2%) | 0,146 | 45 (32.4%) | 8 (72.7%) | 0,0179 | 18 (14.4%) | 16 (44.4%) | <0.001 |
| Reading skills | 11 (8.8%) | 11 (30.6%) | 0,0021 | 27 (12.6%) | 9 (32.1%) | 0,0144 | 7 (14.0%) | 7 (36.8%) | 0,0763 | 23 (16.5%) | 5 (45.5%) | 0,0492 | 11 (8.8%) | 11 (30.6%) | 0,00212 |
| Living conditions | 12 (9.6%) | 4 (11.1%) | 1 | 36 (16.8%) | 4 (14.3%) | 0,945 | 10 (20.0%) | 1 (5.3%) | 0,26 | 34 (24.5%) | 1 (9.1%) | 0,43 | 12 (9.6%) | 4 (11.1%) | 1 |
| Transportation | 15 (12.0%) | 5 (13.9%) | 0,987 | 30 (14.0%) | 4 (14.3%) | 1 | 10 (20.0%) | 9 (47.4%) | 0,0486 | 25 (18.0%) | 8 (72.7%) | <0.001 | 15 (12.0%) | 5 (13.9%) | 0,987 |
| Communication | 1 (0.8%) | 2 (5.6%) | 0,246 | 6 (2.8%) | 1 (3.6%) | 1 | 2 (4.0%) | 3 (15.8%) | 0,243 | 7 (5.0%) | 2 (18.2%) | 0,268 | 1 (0.8%) | 2 (5.6%) | 0,246 |
| Assets | 16 (12.8%) | 2 (5.6%) | 0,36 | 39 (18.2%) | 1 (3.6%) | 0,0906 | 12 (24.0%) | 1 (5.3%) | 0,152 | 35 (25.2%) | 0 (0%) | 0,126 | 16 (12.8%) | 2 (5.6%) | 0,36 |
| Emotional barriers | 1 (0.8%) | 1 (2.8%) | 0,928 | 6 (2.8%) | 1 (3.6%) | 1 | 4 (8.0%) | 2 (10.5%) | 1 | 9 (6.5%) | 2 (18.2%) | 0,405 | 1 (0.8%) | 1 (2.8%) | 0,928 |
| Executive function barriers | 8 (6.4%) | 3 (8.3%) | 0,976 | 21 (9.8%) | 2 (7.1%) | 0,912 | 9 (18.0%) | 4 (21.1%) | 1 | 22 (15.8%) | 3 (27.3%) | 0,575 | 8 (6.4%) | 3 (8.3%) | 0,976 |
| Healthcare navigation barriers | 19 (15.2%) | 9 (25.0%) | 0,264 | 39 (18.2%) | 5 (17.9%) | 1 | 15 (30.0%) | 10 (52.6%) | 0,142 | 35 (25.2%) | 6 (54.5%) | 0,0797 | 19 (15.2%) | 9 (25.0%) | 0,264 |
| Provider attitudes barriers | 10 (8.0%) | 8 (22.2%) | 0,037 | 25 (11.7%) | 5 (17.9%) | 0,53 | 10 (20.0%) | 8 (42.1%) | 0,119 | 25 (18.0%) | 5 (45.5%) | 0,0717 | 10 (8.0%) | 8 (22.2%) | 0,037 |
| Patient-provider communication barriers | 6 (4.8%) | 2 (5.6%) | 1 | 15 (7.0%) | 2 (7.1%) | 1 | 5 (10.0%) | 1 (5.3%) | 0,884 | 14 (10.1%) | 1 (9.1%) | 1 | 6 (4.8%) | 2 (5.6%) | 1 |
| Sensory barriers | 1 (0.8%) | 0 (0%) | 1 | 1 (0.5%) | 0 (0%) | 1 | 3 (6.0%) | 0 (0%) | 0,667 | 3 (2.2%) | 0 (0%) | 1 | 1 (0.8%) | 0 (0%) | 1 |
| SES barriers | 6 (4.8%) | 6 (16.7%) | 0,0425 | 24 (11.2%) | 5 (17.9%) | 0,479 | 5 (10.0%) | 6 (31.6%) | 0,0689 | 23 (16.5%) | 5 (45.5%) | 0,0492 | 6 (4.8%) | 6 (16.7%) | 0,0425 |
| Support barriers | 17 (13.6%) | 5 (13.9%) | 1 | 32 (15.0%) | 4 (14.3%) | 1 | 10 (20.0%) | 4 (21.1%) | 1 | 25 (18.0%) | 3 (27.3%) | 0,72 | 17 (13.6%) | 5 (13.9%) | 1 |
| Waiting room barriers | 6 (4.8%) | 3 (8.3%) | 0,688 | 9 (4.2%) | 3 (10.7%) | 0,303 | 4 (8.0%) | 1 (5.3%) | 1 | 7 (5.0%) | 1 (9.1%) | 1 | 6 (4.8%) | 3 (8.3%) | 0,688 |
| Attending religious services | 13 (10.4%) | 6 (16.7%) | 0,463 | 32 (15.0%) | 3 (10.7%) | 0,754 | 20 (40.0%) | 7 (36.8%) | 1 | 39 (28.1%) | 4 (36.4%) | 0,81 | 13 (10.4%) | 6 (16.7%) | 0,463 |
| Private religious activities | 30 (24.0%) | 5 (13.9%) | 0,286 | 53 (24.8%) | 4 (14.3%) | 0,321 | 24 (48.0%) | 4 (21.1%) | 0,0781 | 47 (33.8%) | 3 (27.3%) | 0,912 | 30 (24.0%) | 5 (13.9%) | 0,286 |
| Intrinsic religiosity | 12 (9.6%) | 5 (13.9%) | 0,667 | 17 (7.9%) | 2 (7.1%) | 1 | 13 (26.0%) | 6 (31.6%) | 0,871 | 18 (12.9%) | 3 (27.3%) | 0,386 | 12 (9.6%) | 5 (13.9%) | 0,667 |
| Hardship | 11 (8.8%) | 5 (13.9%) | 0,56 | 34 (15.9%) | 2 (7.1%) | 0,347 | 10 (20.0%) | 5 (26.3%) | 0,809 | 33 (23.7%) | 2 (18.2%) | 0,961 | 11 (8.8%) | 5 (13.9%) | 0,56 |
| Life stressors | 63 (50.4%) | 24 (66.7%) | 0,125 | 125 (58.4%) | 18 (64.3%) | 0,696 | 36 (72.0%) | 14 (73.7%) | 1 | 98 (70.5%) | 8 (72.7%) | 1 | 63 (50.4%) | 24 (66.7%) | 0,125 |
| Extraversion | 11 (8.8%) | 6 (16.7%) | 0,296 | 31 (14.5%) | 5 (17.9%) | 0,85 | 7 (14.0%) | 5 (26.3%) | 0,395 | 27 (19.4%) | 4 (36.4%) | 0,343 | 11 (8.8%) | 6 (16.7%) | 0,296 |
| Agreeableness | 13 (10.4%) | 7 (19.4%) | 0,245 | 25 (11.7%) | 6 (21.4%) | 0,25 | 10 (20.0%) | 8 (42.1%) | 0,119 | 22 (15.8%) | 7 (63.6%) | <0.001 | 13 (10.4%) | 7 (19.4%) | 0,245 |
| Openness | 22 (17.6%) | 8 (22.2%) | 0,7 | 33 (15.4%) | 10 (35.7%) | 0,0174 | 5 (10.0%) | 2 (10.5%) | 1 | 16 (11.5%) | 4 (36.4%) | 0,061 | 22 (17.6%) | 8 (22.2%) | 0,7 |
| Conscientiousness | 12 (9.6%) | 7 (19.4%) | 0,187 | 28 (13.1%) | 5 (17.9%) | 0,69 | 8 (16.0%) | 6 (31.6%) | 0,27 | 24 (17.3%) | 4 (36.4%) | 0,245 | 12 (9.6%) | 7 (19.4%) | 0,187 |
| Neuroticism | 11 (8.8%) | 7 (19.4%) | 0,137 | 33 (15.4%) | 3 (10.7%) | 0,707 | 8 (16.0%) | 9 (47.4%) | 0,0169 | 30 (21.6%) | 5 (45.5%) | 0,152 | 11 (8.8%) | 7 (19.4%) | 0,137 |
| Life satisfaction | 14 (11.2%) | 7 (19.4%) | 0,311 | 33 (15.4%) | 3 (10.7%) | 0,707 | 16 (32.0%) | 6 (31.6%) | 1 | 35 (25.2%) | 2 (18.2%) | 0,877 | 14 (11.2%) | 7 (19.4%) | 0,311 |
| Coping capability | 17 (13.6%) | 4 (11.1%) | 0,912 | 27 (12.6%) | 5 (17.9%) | 0,636 | 8 (16.0%) | 0 (0%) | 0,152 | 18 (12.9%) | 1 (9.1%) | 1 | 17 (13.6%) | 4 (11.1%) | 0,912 |
| Vigilance | 12 (9.6%) | 6 (16.7%) | 0,376 | 35 (16.4%) | 4 (14.3%) | 0,995 | 11 (22.0%) | 4 (21.1%) | 1 | 34 (24.5%) | 2 (18.2%) | 0,918 | 12 (9.6%) | 6 (16.7%) | 0,376 |
| Everyday discrimination | 6 (4.8%) | 4 (11.1%) | 0,322 | 22 (10.3%) | 4 (14.3%) | 0,75 | 12 (24.0%) | 5 (26.3%) | 1 | 28 (20.1%) | 5 (45.5%) | 0,116 | 6 (4.8%) | 4 (11.1%) | 0,322 |
| Physical activity | 9 (7.2%) | 3 (8.3%) | 1 | 13 (6.1%) | 2 (7.1%) | 1 | 4 (8.0%) | 1 (5.3%) | 1 | 8 (5.8%) | 0 (0%) | 0,904 | 9 (7.2%) | 3 (8.3%) | 1 |
| Sitting | 7 (5.6%) | 0 (0%) | 0,323 | 12 (5.6%) | 0 (0%) | 0,411 | 5 (10.0%) | 1 (5.3%) | 0,884 | 10 (7.2%) | 1 (9.1%) | 1 | 7 (5.6%) | 0 (0%) | 0,323 |
| Smoking | 9 (7.2%) | 3 (8.3%) | 1 | 26 (12.1%) | 2 (7.1%) | 0,642 | 14 (28.0%) | 5 (26.3%) | 1 | 31 (22.3%) | 4 (36.4%) | 0,489 | 9 (7.2%) | 3 (8.3%) | 1 |
| Alcohol | 29 (23.2%) | 5 (13.9%) | 0,33 | 47 (22.0%) | 3 (10.7%) | 0,257 | 18 (36.0%) | 6 (31.6%) | 0,951 | 36 (25.9%) | 4 (36.4%) | 0,688 | 29 (23.2%) | 5 (13.9%) | 0,33 |
| Substance use | 56 (44.8%) | 13 (36.1%) | 0,461 | 90 (42.1%) | 11 (39.3%) | 0,94 | 37 (74.0%) | 11 (57.9%) | 0,314 | 71 (51.1%) | 9 (81.8%) | 0,0983 | 56 (44.8%) | 13 (36.1%) | 0,461 |
| Food insecurity | 9 (7.2%) | 3 (8.3%) | 1 | 31 (14.5%) | 2 (7.1%) | 0,44 | 8 (16.0%) | 7 (36.8%) | 0,122 | 30 (21.6%) | 6 (54.5%) | 0,036 | 9 (7.2%) | 3 (8.3%) | 1 |

Notes: The preclinical Alzheimer’s cognitive composite (PACC) was used to assess the cognitive status of participants.

a CN: Cognitive normal

b Poor cognitive performance (PCP) defined by PACC score below -1 standard deviation from the mean

Supplementary Table 3: Spearman rank correlations between indicators of deprivation (See excel file Supplement table 3)

Supplementary Table 4a Multidimensional poverty measures for Older Black adults with PCI and CN

|  | All Together | | | CN^a^ | | | PCI^b^ | | |  |  |
| --- | --- | --- | --- | --- | --- | --- | --- | --- | --- | --- | --- |
| d | H | A | M0 | H | A | M0 | H | A | M0 | % difference in M0 | P value |
| 1 | 0,98 | 0,17 | 0,17 | 0,98 | 0,16 | 0,16 | 0,99 | 0,20 | 0,19 | 20,67 | 0,02 |
| 2 | 0,91 | 0,18 | 0,17 | 0,90 | 0,17 | 0,16 | 0,93 | 0,21 | 0,19 | 21,03 | 0,02 |
| 3 | 0,80 | 0,20 | 0,16 | 0,78 | 0,19 | 0,15 | 0,86 | 0,22 | 0,19 | 23,29 | 0,02 |
| 4 | 0,71 | 0,22 | 0,15 | 0,68 | 0,21 | 0,14 | 0,75 | 0,24 | 0,18 | 24,07 | 0,03 |
| 5 | 0,58 | 0,24 | 0,14 | 0,55 | 0,23 | 0,13 | 0,63 | 0,26 | 0,17 | 26,81 | 0,03 |
| 6 | 0,46 | 0,27 | 0,12 | 0,44 | 0,25 | 0,11 | 0,50 | 0,30 | 0,15 | 27,33 | 0,05 |
| 7 | 0,38 | 0,30 | 0,11 | 0,34 | 0,28 | 0,10 | 0,44 | 0,32 | 0,14 | 36,41 | 0,02 |
| 8 | 0,30 | 0,32 | 0,10 | 0,27 | 0,31 | 0,08 | 0,37 | 0,34 | 0,13 | 42,04 | 0,02 |
| 9 | 0,26 | 0,34 | 0,09 | 0,23 | 0,32 | 0,07 | 0,32 | 0,36 | 0,12 | 45,46 | 0,02 |
| 10 | 0,21 | 0,36 | 0,08 | 0,18 | 0,34 | 0,06 | 0,27 | 0,38 | 0,10 | 48,18 | 0,02 |
| 11 | 0,16 | 0,39 | 0,06 | 0,13 | 0,37 | 0,05 | 0,23 | 0,40 | 0,09 | 62,42 | 0,01 |
| 12 | 0,13 | 0,41 | 0,05 | 0,10 | 0,39 | 0,04 | 0,19 | 0,42 | 0,08 | 68,26 | 0,01 |
| 13 | 0,10 | 0,43 | 0,04 | 0,07 | 0,42 | 0,03 | 0,16 | 0,44 | 0,07 | 85,43 | 0,00 |
| 14 | 0,08 | 0,45 | 0,04 | 0,05 | 0,45 | 0,02 | 0,14 | 0,45 | 0,06 | 98,92 | 0,00 |
| 15 | 0,06 | 0,48 | 0,03 | 0,04 | 0,47 | 0,02 | 0,10 | 0,48 | 0,05 | 93,62 | 0,01 |
| 16 | 0,05 | 0,49 | 0,03 | 0,03 | 0,48 | 0,02 | 0,09 | 0,50 | 0,04 | 89,44 | 0,02 |
| 17 | 0,03 | 0,54 | 0,02 | 0,01 | 0,55 | 0,01 | 0,06 | 0,53 | 0,03 | 116,90 | 0,02 |
| 18 | 0,03 | 0,54 | 0,02 | 0,01 | 0,55 | 0,01 | 0,06 | 0,53 | 0,03 | 116,90 | 0,02 |
| 19 | 0,02 | 0,56 | 0,01 | 0,01 | 0,58 | 0,01 | 0,04 | 0,55 | 0,02 | 115,96 | 0,02 |
| 20 | 0,02 | 0,57 | 0,01 | 0,01 | 0,58 | 0,01 | 0,03 | 0,57 | 0,02 | 97,13 | 0,11 |
| 21 | 0,01 | 0,59 | 0,01 | 0,01 | 0,58 | 0,01 | 0,01 | 0,62 | 0,01 | 5,30 | 0,50 |
| 22 | 0,01 | 0,61 | 0,00 | 0,00 | 0,59 | 0,00 | 0,01 | 0,62 | 0,01 | 69,33 | 0,31 |
| 23 | 0,00 | 0,62 | 0,00 | 0,00 | NA | NA | 0,01 | 0,62 | 0,01 |  | 0,08 |
| 24 | 0,00 | NA | NA | 0,00 | NA | NA | 0,00 | NA | NA |  | NA |

Notes: The Montreal Cognitive Assessment (MoCA) was used to assess the cognitive status of participants.

a CN: Cognitive normal

b Possible cognitive impairment (PCI) defined by MoCA score equal or below 23

Supplementary Table 4b Multidimensional poverty measures for Older Black adults with PCI and CN by gender

|  | Female CN^a^ | | | Female PCI^b^ | | | | | Male CN^a^ | | | Male PCI^b^ | | | | |
| --- | --- | --- | --- | --- | --- | --- | --- | --- | --- | --- | --- | --- | --- | --- | --- | --- |
| d | H | A | M0 | H | A | M0 | % difference in M0 | P value | H | A | M0 | H | A | M0 | % difference in M0 | P value |
| 1 | 0,96 | 0,15 | 0,14 | 0,99 | 0,17 | 0,17 | 19,58 | 0,03 | 1,00 | 0,22 | 0,22 | 1,00 | 0,27 | 0,27 | 17,53 | 0,16 |
| 2 | 0,88 | 0,16 | 0,14 | 0,92 | 0,18 | 0,17 | 20,23 | 0,03 | 1,00 | 0,22 | 0,22 | 0,96 | 0,27 | 0,26 | 17,14 | 0,16 |
| 3 | 0,72 | 0,18 | 0,13 | 0,85 | 0,19 | 0,16 | 24,18 | 0,03 | 1,00 | 0,22 | 0,22 | 0,88 | 0,29 | 0,26 | 15,56 | 0,16 |
| 4 | 0,61 | 0,20 | 0,12 | 0,73 | 0,21 | 0,16 | 25,16 | 0,04 | 0,93 | 0,23 | 0,22 | 0,81 | 0,31 | 0,25 | 15,65 | 0,15 |
| 5 | 0,47 | 0,22 | 0,11 | 0,57 | 0,24 | 0,14 | 26,49 | 0,07 | 0,84 | 0,25 | 0,21 | 0,81 | 0,31 | 0,25 | 20,25 | 0,12 |
| 6 | 0,38 | 0,24 | 0,09 | 0,42 | 0,28 | 0,12 | 22,95 | 0,16 | 0,68 | 0,27 | 0,19 | 0,73 | 0,33 | 0,24 | 26,93 | 0,10 |
| 7 | 0,30 | 0,27 | 0,08 | 0,35 | 0,30 | 0,11 | 28,64 | 0,12 | 0,50 | 0,31 | 0,16 | 0,69 | 0,34 | 0,24 | 41,21 | 0,05 |
| 8 | 0,23 | 0,29 | 0,07 | 0,29 | 0,33 | 0,10 | 35,66 | 0,09 | 0,43 | 0,33 | 0,14 | 0,62 | 0,36 | 0,22 | 43,40 | 0,06 |
| 9 | 0,20 | 0,30 | 0,06 | 0,25 | 0,34 | 0,09 | 37,12 | 0,10 | 0,34 | 0,36 | 0,12 | 0,54 | 0,38 | 0,21 | 50,02 | 0,06 |
| 10 | 0,14 | 0,33 | 0,05 | 0,22 | 0,36 | 0,08 | 50,68 | 0,05 | 0,34 | 0,36 | 0,12 | 0,42 | 0,42 | 0,18 | 36,05 | 0,17 |
| 11 | 0,10 | 0,35 | 0,04 | 0,19 | 0,37 | 0,07 | 64,42 | 0,03 | 0,23 | 0,41 | 0,09 | 0,35 | 0,45 | 0,16 | 51,43 | 0,12 |
| 12 | 0,07 | 0,37 | 0,03 | 0,16 | 0,38 | 0,06 | 79,79 | 0,01 | 0,20 | 0,42 | 0,09 | 0,27 | 0,50 | 0,13 | 43,71 | 0,20 |
| 13 | 0,05 | 0,39 | 0,02 | 0,13 | 0,40 | 0,05 | 90,42 | 0,01 | 0,14 | 0,47 | 0,06 | 0,27 | 0,50 | 0,13 | 70,93 | 0,08 |
| 14 | 0,03 | 0,42 | 0,01 | 0,11 | 0,41 | 0,05 | 113,87 | 0,01 | 0,11 | 0,49 | 0,06 | 0,23 | 0,52 | 0,12 | 73,31 | 0,09 |
| 15 | 0,02 | 0,43 | 0,01 | 0,06 | 0,43 | 0,03 | 89,50 | 0,07 | 0,09 | 0,52 | 0,05 | 0,23 | 0,52 | 0,12 | 87,31 | 0,06 |
| 16 | 0,02 | 0,43 | 0,01 | 0,04 | 0,45 | 0,02 | 72,99 | 0,18 | 0,09 | 0,52 | 0,05 | 0,23 | 0,52 | 0,12 | 87,31 | 0,06 |
| 17 | 0,00 | NA | NA | 0,01 | 0,49 | 0,01 | NA |  | 0,07 | 0,55 | 0,04 | 0,19 | 0,54 | 0,10 | 94,02 | 0,06 |
| 18 | 0,00 | NA | NA | 0,01 | 0,49 | 0,01 | NA |  | 0,07 | 0,55 | 0,04 | 0,19 | 0,54 | 0,10 | 94,02 | 0,06 |
| 19 | 0,00 | NA | NA | NA | NA | NA | NA | NA | 0,05 | 0,58 | 0,03 | 0,15 | 0,55 | 0,09 | 105,37 | 0,06 |
| 20 | 0,00 | NA | NA | NA | NA | NA | NA | NA | 0,05 | 0,58 | 0,03 | 0,12 | 0,57 | 0,07 | 85,04 | 0,14 |
| 21 | 0,00 | NA | NA | NA | NA | NA | NA | NA | 0,05 | 0,58 | 0,03 | 0,04 | 0,62 | 0,02 | -9,95 | 0,46 |
| 22 | 0,00 | NA | NA | NA | NA | NA | NA | NA | 0,02 | 0,59 | 0,01 | 0,04 | 0,62 | 0,02 | 55,56 | 0,34 |
| 23 | 0,00 | NA | NA | NA | NA | NA | NA | NA | 0,00 | NA | NA | 0,04 | 0,62 | 0,02 | NA |  |
| 24 | 0,00 | NA | NA | NA | NA | NA | 0,00 | NA | NA | NA | NA | NA | NA | NA | NA | NA |

Notes: The Montreal Cognitive Assessment (MoCA) was used to assess the cognitive status of participants; a CN: Cognitive normal; b Possible cognitive impairment (PCI) defined by MoCA score equal or below 23

Supplementary Table 4c Multidimensional poverty measures for Older Black adults with PCI and CN by age groups

|  | Below 65 CN^a^ | | | Below 65 and PCI^b^ | | | | | 65 and above CN^a^ | | | | 65 and above PCI^b^ | | | | | |
| --- | --- | --- | --- | --- | --- | --- | --- | --- | --- | --- | --- | --- | --- | --- | --- | --- | --- | --- |
| d | H | A | M0 | H | A | M0 | % difference in M0 | P value | H | A | M0 | H | | A | M0 | % difference in M0 | P value |  |
| 1 | 1,00 | 0,20 | 0,20 | 1,00 | 0,25 | 0,25 | 25,19 | 0,04 | 0,94 | 0,12 | 0,12 | 0,98 | | 0,15 | 0,15 | 26,04 | 0,01 |  |
| 2 | 0,95 | 0,20 | 0,19 | 0,95 | 0,26 | 0,25 | 25,34 | 0,04 | 0,85 | 0,14 | 0,11 | 0,92 | | 0,16 | 0,15 | 26,95 | 0,01 |  |
| 3 | 0,84 | 0,22 | 0,19 | 0,89 | 0,28 | 0,25 | 27,00 | 0,04 | 0,71 | 0,15 | 0,11 | 0,84 | | 0,17 | 0,15 | 30,70 | 0,01 |  |
| 4 | 0,78 | 0,24 | 0,18 | 0,80 | 0,30 | 0,24 | 26,84 | 0,05 | 0,58 | 0,17 | 0,10 | 0,72 | | 0,19 | 0,14 | 34,37 | 0,02 |  |
| 5 | 0,68 | 0,25 | 0,17 | 0,70 | 0,33 | 0,23 | 28,30 | 0,05 | 0,41 | 0,19 | 0,08 | 0,57 | | 0,21 | 0,12 | 42,72 | 0,02 |  |
| 6 | 0,58 | 0,27 | 0,16 | 0,64 | 0,35 | 0,22 | 32,49 | 0,04 | 0,30 | 0,21 | 0,06 | 0,39 | | 0,25 | 0,10 | 41,32 | 0,06 |  |
| 7 | 0,47 | 0,30 | 0,14 | 0,61 | 0,35 | 0,22 | 42,59 | 0,01 | 0,21 | 0,23 | 0,05 | 0,31 | | 0,27 | 0,08 | 52,00 | 0,05 |  |
| 8 | 0,39 | 0,32 | 0,13 | 0,55 | 0,37 | 0,20 | 46,83 | 0,01 | 0,14 | 0,25 | 0,04 | 0,25 | | 0,29 | 0,07 | 66,09 | 0,03 |  |
| 9 | 0,35 | 0,34 | 0,12 | 0,52 | 0,38 | 0,20 | 52,35 | 0,01 | 0,10 | 0,27 | 0,03 | 0,18 | | 0,31 | 0,06 | 70,90 | 0,05 |  |
| 10 | 0,29 | 0,35 | 0,10 | 0,41 | 0,42 | 0,17 | 50,02 | 0,03 | 0,07 | 0,28 | 0,02 | 0,16 | | 0,32 | 0,05 | 91,08 | 0,02 |  |
| 11 | 0,22 | 0,38 | 0,09 | 0,36 | 0,44 | 0,16 | 60,58 | 0,02 | 0,03 | 0,30 | 0,01 | 0,13 | | 0,33 | 0,04 | 132,41 | 0,01 |  |
| 12 | 0,20 | 0,39 | 0,08 | 0,34 | 0,45 | 0,15 | 65,95 | 0,01 | 0,00 | NA | NA | 0,08 | | 0,36 | 0,03 | NA | 0,00 |  |
| 13 | 0,13 | 0,42 | 0,06 | 0,30 | 0,47 | 0,14 | 84,93 | 0,01 | 0,00 | NA | NA | 0,07 | | 0,36 | 0,02 | NA | 0,00 |  |
| 14 | 0,09 | 0,45 | 0,04 | 0,30 | 0,47 | 0,14 | 105,71 | 0,00 | 0,00 | NA | NA | 0,03 | | 0,38 | 0,01 | NA | 0,03 |  |
| 15 | 0,07 | 0,47 | 0,04 | 0,25 | 0,48 | 0,12 | 109,18 | 0,00 | 0,00 | NA | NA | 0,00 | | NA | NA | NA | NA |  |
| 16 | 0,07 | 0,48 | 0,03 | 0,20 | 0,50 | 0,10 | 105,43 | 0,01 | 0,00 | NA | NA | 0,00 | | NA | NA | NA | NA |  |
| 17 | 0,03 | 0,55 | 0,02 | 0,14 | 0,53 | 0,07 | 129,88 | 0,01 | 0,00 | NA | NA | 0,00 | | NA | NA | NA | NA |  |
| 18 | 0,03 | 0,55 | 0,02 | 0,14 | 0,53 | 0,07 | 129,88 | 0,01 | 0,00 | NA | NA | 0,00 | | NA | NA | NA | NA |  |
| 19 | 0,02 | 0,58 | 0,01 | 0,09 | 0,55 | 0,05 | 129,05 | 0,02 | 0,00 | NA | NA | 0,00 | | NA | NA | NA | NA |  |
| 20 | 0,02 | 0,58 | 0,01 | 0,07 | 0,57 | 0,04 | 112,34 | 0,06 | 0,00 | NA | NA | 0,00 | | NA | NA | NA | NA |  |
| 21 | 0,02 | 0,58 | 0,01 | 0,02 | 0,62 | 0,01 | 26,14 | 0,43 | 0,00 | NA | NA | 0,00 | | NA | NA | NA | NA |  |
| 22 | 0,01 | 0,59 | 0,01 | 0,02 | 0,62 | 0,01 | 87,08 | 0,25 | 0,00 | NA | NA | 0,00 | | NA | NA | NA | NA |  |
| 23 | 0,00 | NA | NA | 0,02 | 0,62 | 0,01 | NA | 0,06 | 0,00 | NA | NA | 0,00 | | NA | NA | NA | NA |  |
| 24 | 0,00 | NA | NA | 0,00 | NA | NA | 0,00 | 24,00 | 0,00 | NA | NA | 0,00 | | NA | NA | 0,00 | NA |  |

Notes: The Montreal Cognitive Assessment (MoCA) was used to assess the cognitive status of participants; a CN: Cognitive normal; b Possible cognitive impairment (PCI) defined by MoCA score equal or below 23

Supplementary Table 4d Multidimensional poverty measures for Older Black adults with PCP and CN

|  | CN^a^ | | | PCP^b^ | | |  |  |  |
| --- | --- | --- | --- | --- | --- | --- | --- | --- | --- |
| d | H | A | M0 | H | A | M0 | P value | % difference in M0 | |
| 1 | 0,977 | 0,167 | 0,163 | 0,979 | 0,212 | 0,207 | 0,011 | 23,558 |  |
| 2 | 0,909 | 0,178 | 0,162 | 0,936 | 0,220 | 0,206 | 0,011 | 24,123 |  |
| 3 | 0,792 | 0,196 | 0,155 | 0,872 | 0,232 | 0,202 | 0,010 | 26,389 |  |
| 4 | 0,691 | 0,213 | 0,147 | 0,787 | 0,248 | 0,196 | 0,011 | 28,351 |  |
| 5 | 0,558 | 0,238 | 0,133 | 0,681 | 0,270 | 0,184 | 0,011 | 32,413 |  |
| 6 | 0,434 | 0,267 | 0,116 | 0,617 | 0,284 | 0,175 | 0,006 | 40,879 |  |
| 7 | 0,347 | 0,293 | 0,102 | 0,532 | 0,304 | 0,162 | 0,006 | 45,413 |  |
| 8 | 0,272 | 0,322 | 0,088 | 0,489 | 0,314 | 0,154 | 0,003 | 54,787 |  |
| 9 | 0,238 | 0,337 | 0,080 | 0,383 | 0,341 | 0,131 | 0,019 | 47,814 |  |
| 10 | 0,185 | 0,364 | 0,067 | 0,362 | 0,347 | 0,125 | 0,007 | 60,255 |  |
| 11 | 0,151 | 0,385 | 0,058 | 0,234 | 0,388 | 0,091 | 0,088 | 43,927 |  |
| 12 | 0,128 | 0,401 | 0,051 | 0,149 | 0,440 | 0,066 | 0,323 | 24,201 |  |
| 13 | 0,094 | 0,428 | 0,040 | 0,128 | 0,459 | 0,059 | 0,230 | 36,887 |  |
| 14 | 0,079 | 0,443 | 0,035 | 0,085 | 0,514 | 0,044 | 0,411 | 21,879 |  |
| 15 | 0,057 | 0,468 | 0,027 | 0,085 | 0,514 | 0,044 | 0,211 | 48,949 |  |
| 16 | 0,045 | 0,484 | 0,022 | 0,085 | 0,514 | 0,044 | 0,122 | 66,358 |  |
| 17 | 0,023 | 0,536 | 0,012 | 0,064 | 0,541 | 0,035 | 0,060 | 95,910 |  |
| 18 | 0,023 | 0,536 | 0,012 | 0,064 | 0,541 | 0,035 | 0,060 | 95,910 |  |
| 19 | 0,015 | 0,561 | 0,008 | 0,043 | 0,568 | 0,024 | 0,104 | 96,188 |  |
| 20 | 0,015 | 0,561 | 0,008 | 0,021 | 0,622 | 0,013 | 0,372 | 43,898 |  |
| 21 | 0,008 | 0,581 | 0,004 | 0,021 | 0,622 | 0,013 | 0,185 | 100,394 |  |
| 22 | 0,004 | 0,595 | 0,002 | 0,021 | 0,622 | 0,013 | 0,082 | 141,983 |  |
| 23 | 0,000 | NaN | NaN | 0,021 | 0,622 | 0,013 | 0,009 | NaN |  |
| 24 | 0 | NaN | NaN | 0 | NaN | NaN | 24 | 0 |  |

Notes: The preclinical Alzheimer’s cognitive composite (PACC) was used to assess the cognitive status of participants; a CN: Cognitive normal; b Poor cognitive performance (PCP) defined by PACC score below -1 standard deviation from the mean

Supplementary Table 4e Multidimensional poverty measures for Older Black adults with PCP and CN by gender

|  | | Female CN^a^ | | | | Female PCP^b^ | | | | | | Male CN^a^ | | | | Male PCP^b^ | | | | |
| --- | --- | --- | --- | --- | --- | --- | --- | --- | --- | --- | --- | --- | --- | --- | --- | --- | --- | --- | --- | --- |
| d | H | | A | M0 | H | | A | M0 | P value | % difference in M0 | H | | A | M0 | H | | A | M0 | P value | % difference in M0 |
| 1 | 0,972 | | 0,153 | 0,148 | 0,964 | | 0,170 | 0,164 | 0,108 | 10,046 | 1,000 | | 0,226 | 0,226 | 1,000 | | 0,270 | 0,270 | 0,090 | 17,716 |
| 2 | 0,888 | | 0,165 | 0,146 | 0,929 | | 0,176 | 0,163 | 0,104 | 10,997 | 1,000 | | 0,226 | 0,226 | 0,947 | | 0,284 | 0,269 | 0,090 | 17,192 |
| 3 | 0,748 | | 0,185 | 0,139 | 0,857 | | 0,186 | 0,159 | 0,100 | 13,916 | 0,980 | | 0,230 | 0,225 | 0,895 | | 0,297 | 0,266 | 0,089 | 16,602 |
| 4 | 0,636 | | 0,204 | 0,129 | 0,786 | | 0,195 | 0,153 | 0,100 | 16,982 | 0,922 | | 0,239 | 0,220 | 0,789 | | 0,326 | 0,257 | 0,085 | 15,489 |
| 5 | 0,491 | | 0,232 | 0,114 | 0,607 | | 0,221 | 0,134 | 0,153 | 16,437 | 0,843 | | 0,251 | 0,212 | 0,789 | | 0,326 | 0,257 | 0,069 | 19,381 |
| 6 | 0,379 | | 0,261 | 0,099 | 0,500 | | 0,239 | 0,120 | 0,162 | 19,288 | 0,667 | | 0,282 | 0,188 | 0,789 | | 0,326 | 0,257 | 0,041 | 31,122 |
| 7 | 0,313 | | 0,281 | 0,088 | 0,357 | | 0,270 | 0,097 | 0,321 | 9,209 | 0,490 | | 0,325 | 0,160 | 0,789 | | 0,326 | 0,257 | 0,021 | 46,983 |
| 8 | 0,238 | | 0,310 | 0,074 | 0,321 | | 0,279 | 0,090 | 0,219 | 19,415 | 0,412 | | 0,351 | 0,145 | 0,737 | | 0,336 | 0,248 | 0,022 | 52,443 |
| 9 | 0,210 | | 0,323 | 0,068 | 0,250 | | 0,297 | 0,074 | 0,347 | 9,151 | 0,353 | | 0,374 | 0,132 | 0,579 | | 0,369 | 0,213 | 0,060 | 47,153 |
| 10 | 0,154 | | 0,351 | 0,054 | 0,250 | | 0,297 | 0,074 | 0,160 | 31,351 | 0,314 | | 0,390 | 0,122 | 0,526 | | 0,381 | 0,201 | 0,071 | 48,394 |
| 11 | 0,131 | | 0,366 | 0,048 | 0,143 | | 0,318 | 0,045 | 0,489 | -5,360 | 0,235 | | 0,430 | 0,101 | 0,368 | | 0,429 | 0,158 | 0,148 | 43,746 |
| 12 | 0,107 | | 0,381 | 0,041 | 0,071 | | 0,338 | 0,024 | 0,256 | -51,616 | 0,216 | | 0,442 | 0,095 | 0,263 | | 0,481 | 0,127 | 0,301 | 28,119 |
| 13 | 0,079 | | 0,401 | 0,032 | 0,036 | | 0,351 | 0,013 | 0,192 | -86,888 | 0,157 | | 0,486 | 0,076 | 0,263 | | 0,481 | 0,127 | 0,165 | 49,567 |
| 14 | 0,065 | | 0,411 | 0,027 | 0,000 | | NA | NA | 0,082 | NA | 0,137 | | 0,506 | 0,069 | 0,211 | | 0,514 | 0,108 | 0,230 | 43,582 |
| 15 | 0,042 | | 0,429 | 0,018 | 0,000 | | NA | NA | 0,135 | NA | 0,118 | | 0,527 | 0,062 | 0,211 | | 0,514 | 0,108 | 0,175 | 54,206 |
| 16 | 0,028 | | 0,441 | 0,012 | 0,000 | | NA | NA | 0,185 | NA | 0,118 | | 0,527 | 0,062 | 0,211 | | 0,514 | 0,108 | 0,175 | 54,206 |
| 17 | 0,005 | | 0,486 | 0,002 | 0,000 | | NA | NA | 0,359 | NA | 0,098 | | 0,546 | 0,054 | 0,158 | | 0,541 | 0,085 | 0,252 | 45,832 |
| 18 | 0,005 | | 0,486 | 0,002 | 0,000 | | NA | NA | 0,359 | NA | 0,098 | | 0,546 | 0,054 | 0,158 | | 0,541 | 0,085 | 0,252 | 45,832 |
| 19 | 0,00 | | NA | NA | 0,00 | | NA | NA | NA | NA | 0,078 | | 0,561 | 0,044 | 0,105 | | 0,568 | 0,060 | 0,362 | 30,385 |
| 20 | 0,00 | | NA | NA | 0,00 | | NA | NA | NA | NA | 0,078 | | 0,561 | 0,044 | 0,053 | | 0,622 | 0,033 | 0,378 | -29,382 |
| 21 | 0,00 | | NA | NA | 0,00 | | NA | NA | NA | NA | 0,039 | | 0,581 | 0,023 | 0,053 | | 0,622 | 0,033 | 0,389 | 35,779 |
| 22 | 0,00 | | NA | NA | 0,00 | | NA | NA | NA | NA | 0,020 | | 0,595 | 0,012 | 0,053 | | 0,622 | 0,033 | 0,225 | 94,909 |
| 23 | 0,00 | | NA | NA | 0,00 | | NA | NA | NA | NA | 0,000 | | NA | NA | 0,053 | | 0,622 | 0,033 | 0,051 | NA |
| 24 | 0,00 | | NA | NA | 0,00 | | NA | NA | NA | NA | 0,000 | | NA | NA | 0,000 | | NA | NA | NA |  |

Notes: The preclinical Alzheimer’s cognitive composite (PACC) was used to assess the cognitive status of participants; a CN: Cognitive normal; b Poor cognitive performance (PCP) defined by PACC score below -1 standard deviation from the mean

Supplementary Table 4f Multidimensional poverty measures for Older Black adults with PCP and CN by age groups

|  | Below 65 CN^a^ | | | Below 65 and PCP^b^ | | | | | 65 and above CN^a^ | | | | 65 and above PCP^b^ | | | | | |
| --- | --- | --- | --- | --- | --- | --- | --- | --- | --- | --- | --- | --- | --- | --- | --- | --- | --- | --- |
| d | H | A | M0 | H | A | M0 | P value | %difference in M0 | H | A | M0 | H | | A | M0 | P value | %difference in M0 |  |
| 1 | 1,000 | 0,203 | 0,203 | 1,000 | 0,332 | 0,332 | 0,004 | 48,366 | 0,952 | 0,126 | 0,120 | 0,972 | | 0,174 | 0,169 | 0,004 | 34,214 |  |
| 2 | 0,950 | 0,212 | 0,201 | 1,000 | 0,332 | 0,332 | 0,004 | 48,995 | 0,864 | 0,136 | 0,117 | 0,917 | | 0,183 | 0,167 | 0,004 | 35,297 |  |
| 3 | 0,843 | 0,232 | 0,195 | 1,000 | 0,332 | 0,332 | 0,004 | 51,731 | 0,736 | 0,150 | 0,110 | 0,833 | | 0,195 | 0,163 | 0,004 | 38,540 |  |
| 4 | 0,771 | 0,246 | 0,190 | 0,909 | 0,357 | 0,324 | 0,004 | 52,442 | 0,600 | 0,165 | 0,099 | 0,750 | | 0,208 | 0,156 | 0,003 | 44,568 |  |
| 5 | 0,671 | 0,266 | 0,179 | 0,909 | 0,357 | 0,324 | 0,004 | 57,866 | 0,432 | 0,188 | 0,081 | 0,611 | | 0,231 | 0,141 | 0,003 | 54,054 |  |
| 6 | 0,571 | 0,289 | 0,165 | 0,909 | 0,357 | 0,324 | 0,003 | 64,984 | 0,280 | 0,216 | 0,061 | 0,528 | | 0,246 | 0,130 | 0,001 | 72,828 |  |
| 7 | 0,479 | 0,314 | 0,150 | 0,909 | 0,357 | 0,324 | 0,002 | 73,393 | 0,200 | 0,238 | 0,048 | 0,417 | | 0,268 | 0,112 | 0,002 | 80,655 |  |
| 8 | 0,400 | 0,338 | 0,135 | 0,909 | 0,357 | 0,324 | 0,001 | 82,234 | 0,128 | 0,265 | 0,034 | 0,361 | | 0,281 | 0,101 | 0,000 | 99,640 |  |
| 9 | 0,371 | 0,348 | 0,129 | 0,727 | 0,392 | 0,285 | 0,006 | 75,266 | 0,088 | 0,287 | 0,025 | 0,278 | | 0,300 | 0,083 | 0,001 | 106,850 |  |
| 10 | 0,300 | 0,373 | 0,112 | 0,636 | 0,413 | 0,263 | 0,008 | 80,669 | 0,056 | 0,313 | 0,018 | 0,278 | | 0,300 | 0,083 | 0,000 | 130,534 |  |
| 11 | 0,250 | 0,393 | 0,098 | 0,455 | 0,470 | 0,214 | 0,037 | 74,031 | 0,040 | 0,330 | 0,013 | 0,167 | | 0,320 | 0,053 | 0,004 | 120,658 |  |
| 12 | 0,229 | 0,402 | 0,092 | 0,364 | 0,514 | 0,187 | 0,075 | 68,078 | 0,016 | 0,378 | 0,006 | 0,083 | | 0,342 | 0,029 | 0,022 | 129,976 |  |
| 13 | 0,164 | 0,432 | 0,071 | 0,364 | 0,514 | 0,187 | 0,026 | 89,760 | 0,016 | 0,378 | 0,006 | 0,056 | | 0,351 | 0,020 | 0,095 | 105,308 |  |
| 14 | 0,136 | 0,450 | 0,061 | 0,364 | 0,514 | 0,187 | 0,013 | 101,502 | 0,016 | 0,378 | 0,006 | 0,000 | | NA | NA | 0,223 | NA |  |
| 15 | 0,107 | 0,468 | 0,050 | 0,364 | 0,514 | 0,187 | 0,005 | 115,259 | 0,000 | NA | NA | 0,000 | | NA | NA | NA | NA |  |
| 16 | 0,086 | 0,484 | 0,042 | 0,364 | 0,514 | 0,187 | 0,002 | 127,259 | 0,000 | NA | NA | 0,000 | | NA | NA | NA | NA |  |
| 17 | 0,043 | 0,536 | 0,023 | 0,273 | 0,541 | 0,147 | 0,001 | 146,071 | 0,000 | NA | NA | 0,000 | | NA | NA | NA | NA |  |
| 18 | 0,043 | 0,536 | 0,023 | 0,273 | 0,541 | 0,147 | 0,001 | 146,071 | 0,000 | NA | NA | 0,000 | | NA | NA | NA | NA |  |
| 19 | 0,029 | 0,561 | 0,016 | 0,182 | 0,568 | 0,103 | 0,006 | 146,239 | 0,000 | NA | NA | 0,000 | | NA | NA | NA | NA |  |
| 20 | 0,029 | 0,561 | 0,016 | 0,091 | 0,622 | 0,057 | 0,124 | 111,638 | 0,000 | NA | NA | 0,000 | | NA | NA | NA | NA |  |
| 21 | 0,014 | 0,581 | 0,008 | 0,091 | 0,622 | 0,057 | 0,038 | 148,768 | 0,000 | NA | NA | 0,000 | | NA | NA | NA | NA |  |
| 22 | 0,007 | 0,595 | 0,004 | 0,091 | 0,622 | 0,057 | 0,009 | 172,039 | 0,000 | NA | NA | 0,000 | | NA | NA | NA | NA |  |
| 23 | 0,000 | NA | NA | 0,091 | 0,622 | 0,057 | 0,000 | NA | 0,000 | NA | NA | 0,000 | | NA | NA | NA | NA |  |
| 24 | 0,000 | NA | NA | 0,000 | NA | NA |  |  | 0,000 | NA | NA | 0,000 | | NA | NA | NA | NA |  |

Notes: The preclinical Alzheimer’s cognitive composite (PACC) was used to assess the cognitive status of participants.; a CN: Cognitive normal; b Poor cognitive performance (PCP) defined by PACC score below -1 standard deviation from the mean

Supplementary Table 5 Indicators contributions to deprivation of S/SDOH by cognitive status, sex and age groups (See Excel file Supplement table 5)

Table 6a. Logistic regression results of area deprivation index on dementia risk-adjusted for age, gender, and marital status.

|  | PCI^a^ | | | PCI^a^ | | | PCP^b^ | | | PCP^b^ | | |
| --- | --- | --- | --- | --- | --- | --- | --- | --- | --- | --- | --- | --- |
|  | Unadjusted model^c^ | | | Adjusted model | | | Unadjusted model^c^ | | | Adjusted model | | |
| *Predictors* | *OR* | *CI* | *p value* | *OR* | *CI* | *p value* | *OR* | *CI* | *p value* | *OR* | *CI* | *p value* |
| (Intercept) | 0.22 | 0.09 – 0.52 | **0.001** | 0.32 | 0.08 – 1.24 | 0.104 | 0.07 | 0.02 – 0.23 | **<0.001** | 0.32 | 0.05 – 1.82 | 0.209 |
| Area Deprivation Index | 1.01 | 1.00 – 1.02 | 0.053 | 1.01 | 1.00 – 1.02 | 0.065 | 1.01 | 1.00 – 1.03 | 0.139 | 1.01 | 1.00 – 1.03 | 0.139 |
| Age (ref: 65 and below) |  |  |  | 1.51 | 0.93 – 2.45 | 0.094 |  |  |  | 4.56 | 2.20 – 10.21 | **<0.001** |
| Sex (ref: male) |  |  |  | 0.75 | 0.43 – 1.34 | 0.331 |  |  |  | 0.25 | 0.12 – 0.52 | **<0.001** |
| Marital status  (ref: Single) |  |  |  | 0.74 | 0.41 – 1.30 | 0.301 |  |  |  | 0.54 | 0.22 – 1.22 | 0.158 |
| Observations | 312 | | | 312 | | | 312 | | | 312 | | |
| R^2^ Tjur^d^ | 0.012 | | | 0.026 | | | 0.007 | | | 0.102 | | |

Notes: OR: odds ratio; CI: confidence interval

The Montreal Cognitive Assessment (MoCA) and the preclinical Alzheimer’s cognitive composite (PACC) used to assess the cognitive status of participants.

a Possible cognitive impairment (PCI) defined by MoCA score equal or below 23

b Poor cognitive performance (PCP) defined by PACC score below -1 standard deviation from the mean

c Unadjusted model includes no covariates

d R2 Tjur pseudo R square.

Table 6b. Logistic regression results of Social Vulnerability Index on Dementia risk adjusted for age, gender, and marital status.

|  | PCI^a^ | | | PCI^a^ | | | PCP^b^ | | | PCP^b^ | | |
| --- | --- | --- | --- | --- | --- | --- | --- | --- | --- | --- | --- | --- |
|  | Unadjusted model^c^ | | | Adjusted model | | | Unadjusted model^c^ | | | Adjusted model | | |
| *Predictors* | *OR* | *CI* | *p value* | *OR* | *CI* | *p value* | *OR* | *CI* | *p value* | *OR* | *CI* | *p value* |

| (Intercept) | | 0.37 | | 0.26 – 0.52 | **<0.001** | 0.48 | 0.15 – 1.47 | 0.206 | 0.12 | 0.07 – 0.20 | **<0.001** | 0.55 | 0.13 – 2.09 | 0.390 | |
| --- | --- | --- | --- | --- | --- | --- | --- | --- | --- | --- | --- | --- | --- | --- | --- |
| Social Vulnerability Index | | 1.14 | | 1.03 – 1.26 | **0.015** | 1.13 | 1.01 – 1.25 | **0.027** | 1.15 | 1.00 – 1.31 | **0.041** | 1.12 | 0.97 – 1.29 | 0.109 | |
| Age (ref: 65 and below) | |  | |  |  | 1.51 | 0.93 – 2.45 | 0.094 |  |  |  | 4.49 | 2.17 – 10.03 | **<0.001** | |
| Sex (ref: male) | |  | |  |  | 0.79 | 0.45 – 1.41 | 0.427 |  |  |  | 0.27 | 0.13 – 0.55 | **<0.001** | |
| Marital status (ref: Single) | |  | |  |  | 0.278 | 0.44 – 1.41 | 0.435 |  |  |  | 0.59 | 0.24 – 1.34 | 0.232 | |
| Observations | | 312 | | | 312 | | | 312 | | | 312 | | |  |  |
| R^2^ Tjur | | 0.020 | | | 0.032 | | | 0.015 | | | 0.102 | | |  |  |

Notes: OR: odds ratio; CI: confidence interval

The Montreal Cognitive Assessment (MoCA) and the preclinical Alzheimer’s cognitive composite (PACC) used to assess the cognitive status of participants.

a Possible cognitive impairment (PCI) defined by MoCA score equal or below 23

b Poor cognitive performance (PCP) defined by PACC score below -1 standard deviation from the mean

c Unadjusted model includes no covariates

d R2 Tjur:pseudo R-square
